# New Crambescidin-Type Alkaloids from the Indonesian Marine Sponge *Clathria bulbotoxa*

**DOI:** 10.3390/md16030084

**Published:** 2018-03-08

**Authors:** Kasmiati Kasmiati, Yukio Yoshioka, Tetsuji Okamoto, Makoto Ojika

**Affiliations:** 1 Graduate School of Bioagricultural Sciences, Nagoya University, Chikusa-ku, Nagoya 464-8601, Japan; kasmiati74@yahoo.com; 2 Faculty of Marine Science and Fishery, Hasanuddin University, Jalan Perintis Kemerdekaan KM. 10, Makassar 90245, Indonesia; 3 Graduate School of Biomedical and Health Sciences, Hiroshima University, Minami-ku, Hiroshima 734-8553, Japan; yyosioka@hiroshima-u.ac.jp (Y.Y.); tetsuok@hiroshima-u.ac.jp (T.O.)

**Keywords:** Indonesian sponge, *Clathria bulbotoxa*, crambescidin, cytotoxicity, anti-oomycete activity

## Abstract

A crude methanolic extract of the Indonesian sponge *Clathria bulbotoxa* showed a potent cytotoxic activity against the human epidermoid carcinoma A431 cells. An investigation of the active components led to the isolation of three new compounds named crambescidins 345 (**1**), 361 (**2**), and 373 (**3**), together with the known related metabolites crambescidins 359 (**4**), 657 (**5**), and 800 (**6**). The structures of the compounds were determined by spectroscopic analysis. These compounds **1**–**4** that possess a simple pentacyclic guanidine core exhibited moderate cytotoxicity against the A431 cells with the IC_50_ values of 7.0, 2.5, 0.94, and 3.1 μM, respectively, while the known compounds **5** and **6** that possess a long aliphatic side chain were found to be significantly cytotoxic. On the other hand, in an anti-oomycete activity test against the fungus-like plant pathogen *Phytophthora capsici*, **1**–**4** showed a higher activity than that of **5** and **6**, suggesting that the long aliphatic side chain plays a significant role for cytotoxicity, but is not effective or suppressive for anti-oomycete activity.

## 1. Introduction

Marine sponges (phyla Porifera) are one of the most prolific and the largest sources of novel bioactive compounds among marine organisms [1,2], with more than 200 new compounds reported each year [3]. During the last decade from 2001 to 2010, approximately 2400 new natural products had been discovered from 671 species of sponges, contributing 29% of the marine natural products reported within the period [4]. Ecological studies reported that sponges produce a wide array of secondary metabolites for defensive purposes to protect them from threats of competitors, predators, and pathogens [5,6]. Furthermore, sponges are frequently a host for microbial symbionts, which are regarded as one of the most important sources of bioactive molecules [7]. 

Studies on Indonesian marine sponges are interesting because Indonesia is the largest archipelagic country in the world, encompassing approximately 86,700 square kilometers of coral reef ecosystems [8], which are important for sponges as the most dominant benthic inhabiting coral reefs [9]. The Indonesian coral reefs are located in the coral triangle area, which is the global center of marine biodiversity and is recognized as the richest region on the earth [10]. Therefore, sponges from the coral ecosystems produce metabolites with various biological properties and become a target of continuing searching for new bioactive compounds [11,12]. 

We recently investigated the biological activity of extracts from Indonesian marine organisms, including six sponge species (*Agelas conifera*, *Carteriospongia foliascens*, *Clathria bulbotoxa*, *Clathria reinwardti*, *Haliclona koromella*, and *Tedania ignis*), and found that an extract of the sponge *C. bulbotoxa* was highly cytotoxic. The sponges of the genus *Clathria* are widely distributed in the tropical shallow waters and temperate regions, especially along the cost of the southern hemisphere [13,14]. This genus has been recognized as an excellent producer of novel secondary metabolites exhibiting diverse chemical structures including alkaloids [15,16,17,18,19,20], carotenoids [21,22], peptides [23], sugars [24], terpenoids [25,26], and sterols [27,28,29]. 

For the above reasons as well as the abundant population of the *Clathria bulbotoxa* in the Samalona Island, South Sulawesi Sea, we were interested in the investigation of bioactive compounds from this sponge, leading to the isolation of three new crambescidin-type guanidine alkaloids **1**–**3** as well as three known related products **4**–**6**. Herein, we present the isolation, structure elucidation, and biological characterization of these guanidine alkaloids.

## 2. Results and Discussion

### 2.1. Isolation and Structural Elucidation

**Figure 1:**
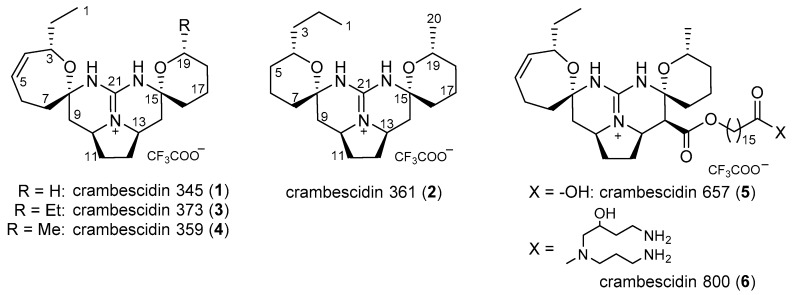
Chemical structures of **1**–**6**.

The MeOH extract of the freeze-dried sponge was found to be higly cytotoxic against the human epidermoid carcinoma cell line A431 at an IC_50_ value of 0.046 μg/mL and to exhibit anti-oomycete activity against the fungus-like plant pathogen *Phytophthora capsici* at a dose of 50 μg/disk. The active extract was subjected to bioassay-guided fractionation, followed by the final reversed-phase high performance liquid chromatography (HPLC) to yield six compounds **1**–**6**. The compounds **4**–**6** were identified as crambescidins 359 [30], 657 [31], and 800 [32] (Figure 1), respectively, by comparison with published spectroscopic data, whereas the compounds **1**–**3** were found to be new crambescidin analogs.

Crambescidin 345 (**1**) possesses the molecular formula of C_20_H_31_N_3_O_2_ deduced from a high resolution electrospry ionization mass spectrum (HR-ESIMS) using the pseudo-molecular ion at *m/z* 346.2495 [M + H]^+^ (calculated for C_20_H_32_N_3_O_2_ 346.2489). The infra red (IR) spectrum of **1** exhibited a characteristic absorption at 3109 cm^−1^, which was also observed for **2** (3107 cm^−1^), **3** (3111 cm^−1^), and the other known crambescidin-type analogs [31,33,34,35]. It was reported that this absorption was due to the N-H streching mode of the cyclic guanidine structure [36]. 

The ^1^H and ^13^C NMR spectra (Table 1 and Table 2) showed that **1** consisted of 30 hydrogen and 20 carbon atoms. A hetero-nuclear single quantum coherence (HSQC) experiment indicated that all of the hydrogen atoms were attached to carbons, revealing the presence of five CH, eleven CH_2_, one CH_3_, and three C. Two additional protons were observed at δ_H_ 10.20 and 10.28 in CDCl_3_ (Figure S2), supporting the presence of the above-mentioned guanidine moiety characteristic of the crambescidin alkaloids. The signals of the three quaternary carbons were found at δ_C_ 85.1 (C-8), 81.3 (C-15), and 149.4 (C-20). Other NMR signals were characterized as an olefinic bond [δ_C_ 134.2/δ_H_ 5.50 (C-4) and δ_C_ 131.4/δ_H_ 5.71 (C-5)], one methyl [δ_C_ 10.8/δ_H_ 0.84 (C-1)], one oxymethylene [δ_C_ 62.6/δ_H_ 3.69 (C-19)], one oxymethine [δ_C_ 72.1/δ_H_ 4.35 (C-3)], two *N*-subtituted methines [δ_C_ 54.9/δ_H_ 4.03 (C-10) and δ_C_ 53.5/δ_H_ 3.96 (C-13)], and ten methylenes (δ_C_ 19.5–39.1/δ_H_ 1.45–2.59) based on their chemical shifts. These NMR data exhibited a close similarity to those for crambescidin 359 (**4**) [30], except for the CH_2_-19 in **1**, which is replaced with an ethylidene (CH_3_-CH<) in **4**.

**Figure 2:**
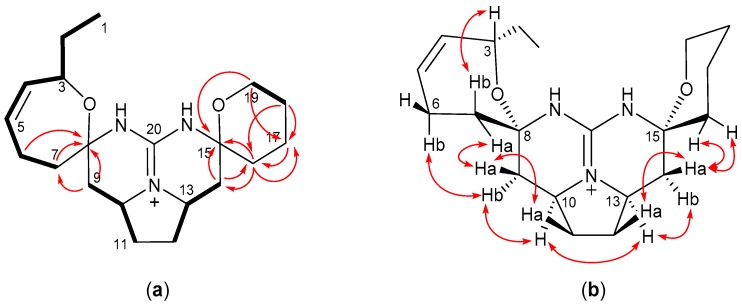
Two dimensional NMR correlations for **1**. (**a**) Key double quantum filtered correlation spectroscopy (DQF-COSY ) (bold bonds), and hetero-nuclear multiple-bond connectivity (HMBC) (solid arrows) correlations; (**b**) Key nuclear Overhauser effect spectroscopy (NOESY ) correlations.

A double quantum filtered correlation spectroscopy (DQF-COSY) experiment was performed to determine the connectivity of the proton-bearing carbons described above, suggesting the presence of four substructures, CH_3_-1–CH_2_-2–CH-3–CH-4=CH-5–CH_2_-6–CH_2_-7, CH_2_-9–CH-10–CH_2_-11, CH_2_-12–CH-13–CH_2_-14, and CH_2_-18–CH_2_-19 (Figure 2a). A hetero-nuclear multiple-bond connectivity (HMBC) experiment was conducted to connect these substructures and the quaternary carbons. The HMBC correlations from H_2_-6, H_2_-7, and H-9 to C-8, and from H-9 to C-7 revealed the position of the quaternary carbon C-8, and the correlations from H_2_-14, H_2_-16, and H_2_-19 to C-15 confirmed the position of the quaternary carbon C-15. The other important HMBC signals were found from H_2_-14, H_2_-17, and H_2_-18 to C-16, from H_2_-16 to C-14, and from H_2_-19, H_2_-18, and H_2_-16 to C-17, supporting the position of the methylene carbons C-16 and C-17. Although the HMBC signal from H-3 to C-8 was not found, the chemical shift of the C-3 (δ_C_ 72.1) indicated that this carbon had an oxygen functionality. Based on these analyses, crambescidin 345 (**1**) was deduced as a new crambescidin analog, lacking the alkyl group at C-19, which is the second example among the crambescidins that were reported previously [35]. 

The relative configuration for **1** was assigned by nuclear Overhauser effect spectroscopy NOESY experiments (Figure 2b). The NOESY correlations of δ_Η_ 4.35 (H-3)/2.27 (H-7b), δ_Η_ 1.97 (H-7a)/1.45 (H-9a), and δ_Η_ 2.42 (H-6b)/2.59 (H-9b) indicated the relative configuration between C-3 and C-8. The relative stereochemistry between C-10 and C-13 was confirmed by the NOESY correlations of H-9a/δ_Η_ 1.75 (H-11a), δ_Η_ 1.75 (H-12a)/1.53 (H-14a), H-9b/δ_Η_ 4.03 (H-10), H-10/δ_Η_ 3.96 (H-13), and H-13/δ_Η_ 2.33 (H-14b). An additional NOESY correlation of δ_Η_ 1.77 (H-16)/H-14a determined the relative configuration between C-15 and other positions. Furthermore, the large coupling constants of H-9a/H-10 (*J* = 12.7 Hz) and H-14a/H-13 (*J* = 13.0 Hz) confirmed the 1,2-diaxial orientation of these hydrogen pairs, concluding the relative stereochemistry of **1,** as described in Figure 2b. The specific rotation value of **1** (−7.1) indicated a close similarity to that of the structurally similar analog **4** (−8.9), suggesting that **1** possesses the same absolute configuration as that of **4** (Figure 1). 

Crambescidin 361 (**2**) possesses the molecular formula of C_21_H_35_N_3_O_2_ deduced from a HR-ESIMS using the pseudo-molecular ion at *m/z* 362.2765 [M + H]^+^ (calculated for C_21_H_36_N_3_O_2,_ 362.2802). Detailed analysis of the ^1^H and ^13^C NMR spectral data (Table 1 and Table 2) indicated that **2** consisted of 34 hydrogene and 21 carbon atoms, and HSQC experiments implied that all the protons were connected to carbons (four CH, twelve CH_2_, two CH_3_, and three C). In addition, two exchangeable proton signals were observed at δ_Η_ 10.13, and 10.16 in acetone-*d*_6_ (Figure S17 and Table S1). The signals of the three quaternary carbons were observed at δ_C_ 81.6 (C-8 and C-15) and 149.0 (C-21). Other important NMR signals were recognized as two methyls [δ_C_ 13.8/δ_H_ 0.87 (C-1) and δ_C_ 22.0/δ_H_ 1.11 (C-20)], two oxymethines [δ_C_ 71.1/δ_H_ 3.63 (C-4) and δ_C_ 68.2/δ_H_ 3.74 (C-19)], two *N*-subtituted methines [δ_C_ 53.7/δ_H_ 4.00 (C-10) and δ_C_ 53.4/δ_H_ 4.00 (C-13)], and twelve methylenes [δ_C_ 19.4–40.4/δ_H_ 1.26–3.30]. Although these data were characteristic of the crambescidin alkaloids, they showed the absence of the olefinic function that was found in most of the reported crambescidin-type alkaloids. In addition, the pentacyclic guanidine core is symmetrical, as indicated by highly overlapping chemical shifts of the corresponding proton and carbon signals (Table 1 and Table 2). 

**Figure 3:**
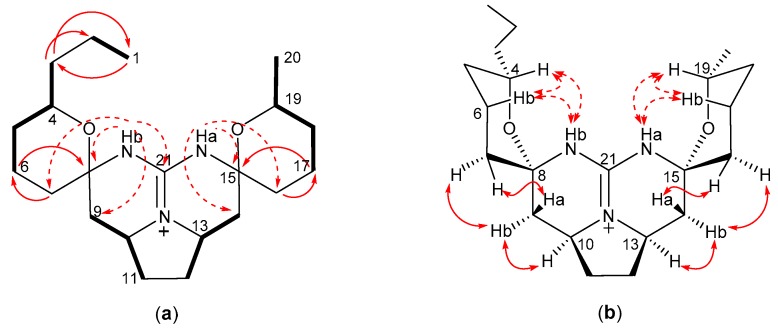
Two dimensional NMR correlations for **2**. (**a**) Key DQF-COSY (bold bonds) and HMBC correlations (solid arrows in CD_3_OD and dotted arrows in acetone-*d*_6_); (**b**) Key NOESY correlations in CD_3_OD (solid arrows) and acetone-*d*_6_ (dotted arrows). The position of the propyl and methyl groups at C-4 and C-19 are tentative.

The DQF-COSY correlations for **2** indicated the five partial structures CH_3_-1–CH_2_-2, CH_2_-3–CH-4–CH_2_-5–CH_2_-6, CH_2_-9–CH-10–CH_2_-11, CH_2_-12–CH-13–CH_2_-14, and CH_2_-17–CH_2_-18–CH-19–CH_3_-20 (Figure 3a). The HMBC correlations from H_3_-1 to C-3, and from H_2_-3 to C-2 and C-1 confirmed the presence of a propyl group on C-4. Furthermore, the correlations from H_2_-6 to C-8, from H_2_-7 to C-6, from H_2_-17 to C-15, and from H_2_-16 to C-17 confirmed the connectivities of C-6–C-7 and C-16–C-17. An HMBC spectrum was obtained in acetone-*d*_6_ (Figure 3a, dotted arrows), indicating the following important correlations: from NHb to C-7, C-8, C-9, and C-21 and from NHa to C-14, C-15, and C-16, revealing not only the presence of guanidine moiety, but also the position of C-7, C-8, C-15, and C-16. Based on these analyses, crambescidin 361 (**2**) was deduced as a new crambescidin analog with two tetrahydropyrane rings instead of the combination of the left-side unsaturated seven membered ring and the right-side tetrahydropyrane ring as found in **1** and most of the crambescidin analogs. Another structural feature of **2** is the presence of a propyl group as an alkyl substituent, which is quite rare in the crambescidin-type alkaloids [37,38].

The relative configuration of **2** was examined by the interpretation of NOESY correlations (Figure 3b). The chair conformation of both the tetrahydropyrane rings was determined by the 1,3-diaxial correlations of δ_Η_ 3.63 (H-4)/1.85 (H-6b) and δ_Η_ 3.74 (H-19)/1.85 (H-17b) (Figure 3b, dotted double arrows). The NOESY correlations of δ_Η_ 10.16 (NHb)/H-4, NHb/H-6b, δ_Η_ 10.13 (NHa)/H-19, and NHa/H-17b indicated that both the guanidine NHs were also in the axial orientation about the tetrahydropyrane rings. The additional NOESY correlations of δ_Η_ 1.74 (H-7)/1.57 (H-9a), H-7/2.19 (H-9b), H-9b/δ_Η_ 4.00 (H-10), δ_Η_ 4.00 (H-13)/2.21 (H14b), δ_Η_ 1.59 (H14a)/1.74 (H-16), and H14b/H-16 suggested the relative configuration between C-8 and C-10 and between C-13 and C-15. Although the NOESY correlation of H-10/H-13 was not obtained due to their identical chemical shifts, the large coupling constants of 12.8 Hz between H-9a and H-10 and between H-14a and H-13 described that both H-10 and H-13 were in the axial-like α orientation. These findings support the relative stereochemistry of **2**, as shown in Figure 3b. Since it was difficult to determine the position of two alkyl groups due to the highly symmetrical nature of **2**, we tentatively assigned the alkyl position as shown becasue most of the related guanidine alkaloids possess a methylated tetrahydropyrane ring at the right side of the molecule. The specific rotation of **2** (−7.9) similar to that of **1** suggests the identical absolute configuration of **1** and **2**. 

**Figure 4:**
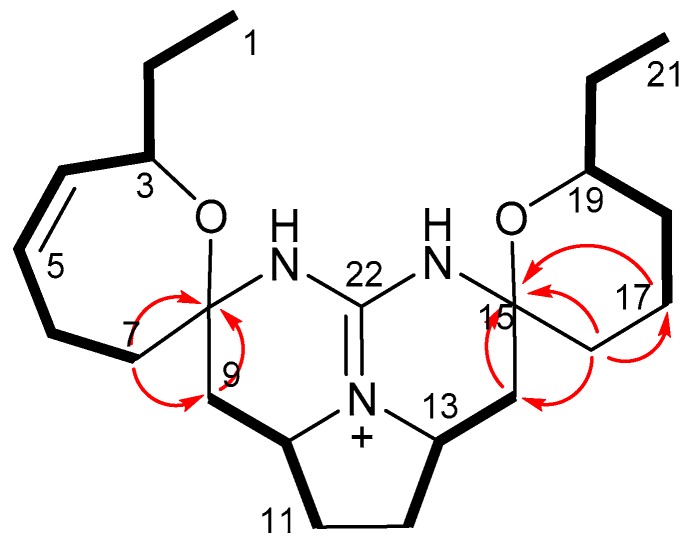
Two dimensional NMR correlations of **3**. DQF-COSY and HMBC correlations are indicated by bold bonds and arrows, respectively.

Crambescidin 373 (**3**) possesses the molecular formula C_22_H_35_N_3_O_2_ as determined by the pseudo-molecular ion at *m*/*z* 374.2786 [M + H]^+^ (calculated for C_22_H_36_N_3_O_2_, 374.2802) in HR-ESIMS. The NMR data (Table 1 and Table 2) were found to be similar to those for **1** and **4**, indicating that **3** was another crambescidin analog with an ethyl group as supported by the signals at δ_H_ 1.42/δ_C_ 30.0 (CH_2_-20) and δ_H_ 0.85/δ_C_ 10.2 (CH_3_-21). This ethyl group was found to be connected to C-19 by a DQF-COSY experiment (Figure 4). The exchangeable protons of **3** were observed at δ_H_ 10.49 and 10.06 in CDCl_3_, revealing that **3** is the 19-ethyl homolog of **1**. Although satisfactory NOESY data for **3** was not obtained due to the lack of the sample, the close similarity of the NMR data and the specific rotation (−8.0 for **3** and −8.9 for **4**) to those for **4** suggests that the absolute configuration of **3** is the same as that of **4**. 

Guanidine compounds were mostly reported from marine organisms [39]. They have intriguing structures and wide range of biological activities and have attracted much attention of chemists and pharmacologists for their potential as drug leads [40]. Due to the strongest organic bases, guanidines are fully protonated under physiological conditions to form guanidinium cation, which can interact with biopolymers, such as DNA and proteins through hydrogen bonds and/or electrostatic interactions [41,42]. Since the first pentacyclic guanidine alkaloid ptilomycalin A was isolated from the Caribbean sponge *Ptilocaulis spiculifer* and a Red Sea sponge *Hemimycale* sp. in 1989 [43], an array of cyclic guanidine alkaloids has been reported to date, including ptilomycalins [44,45], crambescidins [30,32,33,35,46,47], monanchocidins [48,49], and monanchomycalins [37,38]. Particularly, a number of metabolites of these types have been isolated mainly from marine sponges of the genera *Monanchora* and *Crambe*. The crambescidins and related alkaloids are characterized by a pentacyclic guanidine skeleton (vessel part) with two alkyl groups (ethyl at C-3 and methyl at C-19 in most cases) and a long aliphatic chain with a terminal carboxylate or a terminal spermidine amide. Crambescidins 359 (**4**) and 431 were reported in 2000 as the first crambescidin analogs lacking the long aliphatic chain (at C-14 in **5** and **6**), which is replaced by a hydrogen atom and an ethyl ester group, respectively [30]. Our compounds **1**–**3** are additional analogs of this type and are structurally characteristic in the following points. Crambescidin 345 (**1**) is the first analog with a non-alkylated tetrahydropyrane ring. Crambescidin 361 (**2**) possesses a rare propyl substituent as well as two tetrahydropyrane rings instead of the combination of one unsaturated oxepane and one tetrahydropyrane rings, which are found in most of the crambescidin-type alkaloids. Crambescidin 373 (**3**) is the first analog with an ethyl group at the right-side tetrahydropyrane ring, which possesses a methyl group in most of the reported crambescidin-type alkaloids. 

### 2.2. Biological Activity 

**Figure 5:**
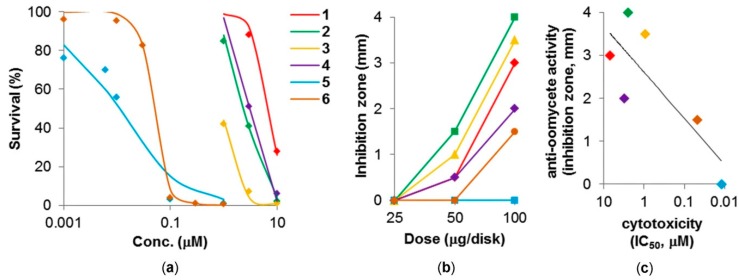
Biological activity of **1**–**6**. (**a**) Cytotoxicity against the human epidermoid carcinoma A431 cells. The curves were generated by sigmoid fitting; (**b**) Anti-oomycete activity against the plant pathogen *P. capsici*. The same colors are used as in Figure 5a; (**c**) Inverse relationship between the cytotoxicity and anti-oomycete activities of **1**–**6**. The data for anti-oomycete activity were observed at a dose of 100 μg/disk.

Biological activities of the isolated crambescidins **1**–**6** were evaluated as cytotoxic and anti-oomycete agents against the human epidermoid carcinoma cell line A431 and the oomycete plant pathogen *Phytophthora capsici*, respectively. All of the compounds showed cytotoxicity with an IC_50_ value lower than 10 μM (Figure 5a). The strongest cytotoxicity was observed for the long side chain-bearing crambescidins 5 and 6 with IC_50_’s of 12 and 48 nM, respectively. Meanwhile, other crambescidins (**1**–**4**) without the long side chain part indicated a moderate cytotoxicity with IC_50_’s of 7.0, 2.5, 0.94, and 3.1 μM, respectively. On the contrary, all new crambescidins **1**–**3** and the known analog 4 showed a higher anti-oomycete activity [minimum inhibitory dose (MID) of 50 μg/disk] than that for 5 and 6 (MID of 100 μg/disk or higher) (Figure 5b). It is interesting to note that the highly cytotoxic crambescidins (5 and 6) with a long side chain showed a lower anti-oomycete activity than the others. Especially, the most cytotoxic compound 5 showed no anti-oomycete activity even at 500 μg/disk (not indicated in Figure 5b). Consequently, these biological activities are approximately in an inverse relationship (Figure 5c).

Previous structure-activity relationship (SAR) studies on the crambescidins and their analogs reported that the presence of the long aliphatic side chain enhanced the cytotoxic effect of the guanidine core [50,51,52,53]. Our cytotoxicity data indicating the significant effect of the long aliphatic side chain are in good agreement with the previous reports. The long aliphatic side chain could affect the permeability of the guanidine alkaloid into animal cells. In contrast, it did not affect or rather diminished the anti-oomycete activity, which might be attributable to a low permeability through the microbial cell wall (mainly β-glucan) or/and into the hydrophilic agar medium that is used in the test.

## 3. Materials and Methods 

### 3.1. General Procedures

Thin layer chromatography (TLC) was conducted by using precoated silica gel 600 F254 plates (Art. 5715, Merck, Darmstadt, Germany) or reverse phase C18 F254 plates (Art. 15389, Merck, Darmstadt, Germany). Flash column chromatography was carried out on silica gel by a medium-pressure gradient system equipped with a Pump Module C-605 and a Pump Manager C-615 (BÜCHI, Flawil, Switzerland). High resolution ESIMS were recorded on a Mariner Biospectrometry Workstation (Applied Biosystems of Thermo Fisher Scientific, Waltham, MA, USA) equipped with an electrospray ion source in the positive mode. High performance liquid chromatography (HPLC) was performed on a high pressure gradient system that is composed of pumps PU-2087, a degasser DG-2080-53, a mixer MX-2080-32, and a detector UV-2075 (JASCO, Tokyo, Japan). FT-IR spectra were recorded on an FT/IR-400 spectrometer instrument (JASCO). Specific rotations were observed on a DIP-370 polarimeter (JASCO). NMR spectra were investigated on an Avance ARX400 (400 MHz for ^1^H) or Avance III HD 600 MHz Cryo-probe spectrometer (600 MHz for ^1^H) (Bruker Bio Spin, Yokohama, Japan). The chemical shifts (ppm) were referenced to the solvent residual peak at δ_H_ 7.26 ppm (CDCl_3_), δ_H_ 3.30/δ_C_ 49.0 ppm (CD_3_OD), or δ_H_ 2.06/δ_C_ 29.9 ppm (acetone-*d*_6_). 

### 3.2. Isolation of Bioactive Compounds

A reddish Indonesian sponge was collected by hand using a snorkeling equipment at a depth between 0.5 and 3 m in Samalona Island (5°8′16.4″ S–119°23′22.60″ E), South Sulawesi Sea, in August 2015. The species was identified as *Clathria bulbotoxa* based on the observation of its morphology and spicule elements under a microscope. The sponge possesses bulbous toxa spicule bulging toward the center that distinguishes it from other species [54]. The organism (750 g, wet weight) was lyophilized, homogenized in MeOH (1.5 L) and stand at room temperature for 3 days. The mixture was filtrated and the filtrate was concentrated to give an aqueous residue, which was extracted three times with EtOAc (225 mL). The combined organic layers were concentrated to yield EtOAc extract (6.4 g). This extract was dissolved in 90% MeOH (75 mL) and extracted twice with hexane (150 mL). Both of the layers were concentrated to obtain 90% MeOH (3.1 g) and hexane (2.5 g) fractions. 

The 90% MeOH fraction, which showed a cytotoxicity (IC_50_ = 0.046 μg/mL), was separated by open column chromatography (silica gel, 100 g) eluted with 2, 5, 10, 100% of MeOH in CHCl_3_ (600 mL each). The fractions were collected by every 100 mL, and appropriately combined to give six fractions (fr.1–fr.6), where fr.3 (222 mg) eluted with 2–5% MeOH and fr.6 (2.1 g) eluted with 100% MeOH showed significant cytotoxic activity of IC_50_ = 0.013 and 0.092 μg/mL, respectively. Fr.3 was further chromatographed on silica gel (HI-FLASH^TM^ Size L, 30 g, Yamazen Co., Osaka, Japan) with linear gradient of 10–80% CHCl_3_-MeOH-H_2_O (90:9:1) in EtOAc (40 min) at a flow rate of 10 mL/min to yield eight fractions (fr.3-1–fr.3-8). The cytotoxic fr.3-5 (33.1 mg, IC_50_ = 0.8 ng/mL) was purified by HPLC [Develosil ODS-HG-5 (20 × 250 mm, Nomura Chemical Co., Ltd., Seto, Aichi, Japan), 80–100% MeOH-20 mM NH_4_CH_3_COO (40 min), 6 mL/min, detected at 215 nm] to give crambescidin 657 (**5**, 16.2 mg, *t*_R_ = 48.0 min). The three fractions, fr.3-6–fr.3-8 (110 mg in total, IC_50_ = 0.081 μg/mL) were combined and subjected to HPLC [Develosil ODS-HG-5 (20 × 250 mm), 50–80% MeOH-0.1% trifluoroacetic acid (TFA) (60 min), flow rate 6 mL/min, monitored at 205 nm] to obtain a **1**-containing fraction (1.5 mg, *t*_R_ = 40.8 min), crambescidin 359 (**4**, 26.0 mg, *t*_R_ = 45.6 min), crambescidin 361 (**2**, 2.4 mg, *t_R_* = 55.4 min), and crambescidin 373 (**3**, 1.1 mg, *t*_R_ = 62.0 min). The **1**-containing fraction was further purified by HPLC [Develosil ODS-UG-5 (10 × 250 mm), 60% MeOH-0.1% TFA, 2 mL/min, detected at 215 nm] to obtain pure crambescidin 345 (**1,** 0.6 mg, *t_R_* = 19.7 min). The fr.6 (2.1 g, IC_50_ = 0.092 μg/mL) was fractionated through a silica gel (50 g) open column, eluted with 10, 20, 40, 60, 100% MeOH-H_2_O (90:10) in CHCl_3_ to afford four fractions (fr.6-1–fr.6-4). The active fr.6-3 (500 mg, IC_50_ = 0.017 μg/mL) eluted with 40% MeOH-H_2_O (90:10) in CHCl_3_ was then chromatographed on silica gel (HI-FLASH^TM^, Size L, 30 g) with gradient elution of 10–100% MeOH in CHCl_3_ for 40 min at a flow rate of 10 mL/min to give four fractions (fr.6-3-1–fr.6-3-4). The active fr. 6-3-3 (180 mg, IC_50_ = 0.019 μg/mL) eluted with 54–86% MeOH was subjected to HPLC [Develosil ODS-HG-5 (20 × 250 mm), 40–60% MeCN-0.1% TFA (60 min), 8 mL/min, detected at 230 nm] to obtain crambescidin 800 (**6**, 21.7 mg, *t_R_* = 36.3 min). 

#### 3.2.1. Crambescidin 345 (**1**)

Colorless powder; [α${]}_{D}^{26}$ −7.1 (0.051, MeOH); IR (film) *ν*_max_ 3222, 3109, 3019, 1678, 1654, 1607, 1201, 1177, 1131, and 720 cm^−1^; HR ESIMS *m/z* 346.2495 [M + H]^+^; calcd. for C_20_H_32_N_3_O_2_ 346.2489. 

#### 3.2.2. Crambescidin 361 (**2**)

Pale yellow solid; [α${]}_{D}^{26}$ −7.9 (0.13, MeOH); IR (film) *ν*_max_ 3236, 3107, 1676, 1652, 1606, 1201, 1176, 1131, 1017, and 719 cm^−1^; HR ESIMS *m/z* 362.2765 [M + H]^+^; calcd. for C_21_H_36_N_3_O_2_ 362.2802.

#### 3.2.3. Crambescidin 373 (**3**)

Pale yellow solid; [α${]}_{D}^{26}$ −8.8 (0.025, MeOH); IR (film) *ν*_max_ 3228, 3111, 3019, 1678, 1652, 1606, 1201, 1177, 1131, and 720 cm^−1^; HR ESIMS *m/z* 374.2786 [M + H]^+^; calcd. for C_22_H_36_N_3_O_2_ 374.2802.

#### 3.2.4. Crambescidin 359 (**4**)

Pale yellow solid; [α${]}_{D}^{26}$ −8.9 (0.23, MeOH) (reference [45]: [α${]}_{D}^{20}$ −12.7 (0.4, MeOH)).

#### 3.2.5. Crambescidin 657 (**5**)

Yellowish solid; [α${]}_{D}^{25}$ −11.0 (0.18, MeOH) (reference [55]: [α${]}_{D}^{25}$ −12.1 (0.34, MeOH)).

#### 3.2.6. Crambescidin 800 (**6**)

Yellowish solid; [α${]}_{D}^{25}$ −8.7 (1.6, MeOH) (reference [45]: [α${]}_{D}^{20}$ −7.8 (4.1, MeOH)).

### 3.3. Anti-oomycetete Assay

The test was performed by the paper disk diffusion method [56]. Briefly, a piece of the mycelia of the plant pathogen *Phytophthora capsici* NBRC 30696 was pre-cultured on a potato-glucose-agar medium in a 9-cm dish at 25 °C and 60% humidity for seven days in the dark. A piece (5 × 5 mm) of the colony was then inoculated on the center of a 5% V8 juice-1.5%-agar medium in a 9-cm dish and incubated for 48 h at 25 °C and 60% humidity. A paper disk (8 mm in diameter) containing a sample at an appropriate dose (none, 25, 50, and 100 μg/disk) was placed at 1 cm away from the colony front. After incubation for another 22–24 h, the inhibition zone formed around the sample disk was measured. The activity was represented by the minimum dose that expressed an obvious inhibition zone (usually 0.5 mm or wider).

### 3.4. Cytotoxicity Assay

A431 human vulva-derived epidermoid carcinoma cells were used to evaluate the cytotoxicity of the compounds under the conditions reported previously [57]. Briefly, the cells at a density of 1.0 × 10^4^ cells/well were cultured in a 24-well plate (BD Falco) in DF6F medium with various concentrations of compounds (none, 1, 3, 10 μM for **1**–**4**; none, 0.001, 0.01, 0.1, and 1.0 μM for **5** and **6**) at 37 °C in a humidified 95% air/5% CO_2_ condition in a CO_2_ incubator (Thermo Fisher Scientific, Waltham, MA, USA), followed by cell counting with a Coulter Counter (Coulter Electronics Inc., Hialeah, FL, USA) on day 5. The DF6F medium was composed of a 1:1 ratio of DMEM and Ham F-12 medium (DF), supplemented with six factors, i.e., insulin (10 μg/mL), transferrin (5 μg/mL), 2-aminoethanol (10 μM), sodium selenite (10 nM), 2-mercaptoethanol (10 μM), and oleic acid conjugated with fatty acid-free bovine serum albumin (9.4 μg/mL) (all of the chemicals were from Sigma-Aldrich, St. Louis, MO, USA). IC_50_ values are shown by the means of two (for **1**–**4** and **6**) or three (**5**) replicates. 

## 4. Conclusions

In the present study, we discovered three new guanidine alkaloids, crambescidins 345 (**1**), 361 (**2**), and 373 (**3**), together with three known crambescidins **4**–**6** from the Indonesian sponge *Clathria bulbotoxa*. The structures of **1**–**3** with absolute stereochemistry were determined by spectroscopic analysis, including two-dimensional NMR and specific rotation. Although the pentacyclic guanidine core has been found in a number of the crambescidins and related natural compounds, a high diversity in the alkyl substituents (methyl, ethyl, propyl) on the cyclic ether rings of our compounds was observed for the first time, whereas most related products possess ethyl group at C-3 and methyl group at C-19. The biological assays revealed that the long aliphatic side chain in compounds **5** and **6** plays a quite important role for the cytotoxicity against cancer cells (possibly due to the increase of permeability through cell membrane), but conversely not for the inhibition of an oomycete plant pathogen. 

## Figures and Tables

**Table 1:** ^1^H NMR data for **1**–**3** (CD_3_OD).

Position	1 ^a^	2 ^b^	3 ^a^
1	0.84, t (7.2)	0.87, t (6.8)	0.84, t (7.2)
2a	1.46, m	1.38, m	1.46, m
2b	1.54, m		1.54, m
3	4.35, brd (10.8)	1.41, m	4.33, brd (10.2)
		1.47, m	
4	5.50, dt (10.8, 2.1)	3.63, brt (12.6)	5.50, dt (11.2, 2.1)
5a	5.71, m	1.29, m	5.71, m
5b		1.67, m	
6a	2.15, dt (15.3, 7.2)	1.74, m	2.15, dt (15.3, 7.2)
6b	2.42, brt (15.3)	1.85, m	2.42, brt (15.3)
7a	1.97, dd (13.5, 6.0)	1.74, m	1.97, dd (13.5, 6.0)
7b	2.27, t (13.5)		2.27, t (13.5)
9a	1.45, t (12.7)	1.57, t (12.8) ^c^	1.45, t (12.9)
9b	2.59, dd (12.7, 4.8)	2.19, dd (12.8, 4.2) ^d^	2.59, dd (12.9, 4.8)
10	4.03, m	4.00, m	4.05, m
11a	1.75, m	1.73, m	1.75, m
11b	2.31, m	2.30, m	2.32, m
12a	1.75, m	1.73, m	1.75, m
12b	2.31, m	2.30, m	2.32, m
13	3.96, m	4.00, m	4.03, m
14a	1.53, t (13.0)	1.59, t (12.8) ^c^	1.59, t (13.0)
14b	2.33, dd (13.0, 4.5)	2.21, dd (12.8, 4.2) ^d^	2.21, dd (13.0, 4.8)
16a	1.77, m	1.74, m	1.73, m
16b			1.77, m
17a	1.80, m	1.74, m	1.77, m
17b		1.85, m	1.85, m
18a	1.61, m	1.26, m	1.26, m
18b		1.70, m	1.70, m
19	3.69, m	3.74, m	3.50, m
20		1.11, d (9.0)	1.42, m
21			0.85, t (7.2)

Data were observed at ^a^ 600 MHz or ^b^ 400 MHz; ^c,d^ Interchangeable signal within the same marks.

**Table 2:** ^13^C NMR data for **1**–**3** (CD_3_OD).

Position	1 ^a^	2 ^b^	3 ^a^
1	10.8, CH_3_	13.8, CH_3_	11.3, CH_3_
2	30.3, CH_2_	19.4, CH_2_	30.3, CH_2_
3	72.1, CH	38.7, CH_2_	72.1, CH
4	134.2, CH	71.1, CH	134.3, CH
5	131.4, CH	31.7, CH_2_	131.4, CH
6	24.4, CH_2_	19.5, CH_2_^c^	24.5, CH_2_
7	38.5, CH_2_	34.7, CH_2_^d^	38.5, CH_2_
8	85.1, C	81.6, C	85.1, C
9	37.9, CH_2_	40.4, CH_2_	37.9, CH_2_
10	54.9, CH	53.7, CH ^e^	54.9, CH
11	30.8, CH_2_	30.7, CH_2_	30.8, CH_2_
12	30.8, CH_2_	30.7, CH_2_	30.8, CH_2_
13	53.5, CH	53.4, CH^e^	53.5, CH
14	39.1, CH_2_	40.4, CH_2_	40.4, CH_2_
15	81.3, C	81.6, C	81.5, C
16	35.1, CH_2_	34.6, CH_2_^d^	34.7, CH_2_
17	19.5, CH_2_	19.7, CH_2_^c^	19.5, CH_2_
18	25.9, CH_2_	33.3, CH_2_	31.3, CH_2_
19	62.6, CH_2_	68.2, CH	73.3, CH
20	149.4, C	22,0 CH_3_	30.0, CH_2_
21		149.0, C	10.2, CH_3_
22			149.4, C

Data were obtained at ^a^ 150 MHz or ^b^ 100 MHz; The number of hydrogen on carbon was determined by a hetero-nuclear single quantum coherence (HSQC); ^c–e^ Interchangeable signals within the same marks.

## Supplementary Materials

The following are available online at http://www.mdpi.com/1660-3397/16/3/84/s1Table S1: ^1^H and ^13^C NMR data of **2** (600 MHz, acetone-*d*_6_), Figure S1: ^1^H NMR spectrum of **1** (600 MHz, CD_3_OD), Figure S2: ^1^H NMR spectrum of **1** (400 MHz, CDCl_3_), Figure S3: ^13^C NMR spectrum of **1** (150 MHz, CD_3_OD), Figure S4: DQF-COSY spectrum of **1** (600 MHz, CD_3_OD), Figure S5: HSQC spectrum of **1** (600 MHz, CD_3_OD), Figure S6: HMBC spectrum of **1** (600 MHz, CD_3_OD), Figure S7: NOESY spectrum of **1** (600 MHz, CD_3_OD), Figure S8: IR spectrum of **1**, Figure S9: ESI-TOF-MS(+) spectra of **1**–**3**, Figure S10: ^1^H NMR spectrum of **2** (400 MHz CD_3_OD), Figure S11: ^13^C NMR spectrum of **2** (100 MHz, CD_3_OD), Figure S12: DQF-COSY spectrum of **2** (400 MHz, CD_3_OD), Figure S13: HSQC spectrum of **2** (400 MHz, CD_3_OD), Figure S14: HMBC spectrum of **2** (400 MHz, CD_3_OD), Figure S15: NOESY spectrum of **2** (400 MHz, CD_3_OD), Figure S16: IR spectrum of **2**, Figure S17: ^1^H NMR spectrum of **2** (600 MHz acetone-*d*_6_), Figure S18: ^13^C NMR spectrum of **2** (150 MHz, acetone-*d*_6_), Figure S19: DQF-COSY spectrum of **2** (600 MHz, acetone-*d*_6_), Figure S20: HSQC spectrum of **2** (600 MHz, acetone-*d*_6_), Figure S21: HMBC spectrum of **2** (600 MHz, acetone-*d*_6_), Figure S22: NOESY spectrum of **2** (600 MHz, acetone-*d*_6_), Figure S23: ^1^H NMR spectrum of **3** (600 MHz, CD_3_OD), Figure S24: ^1^H NMR spectrum of **3** (400 MHz, CDCl_3_), Figure S25: ^13^C NMR spectrum of **3** (150 MHz, CD_3_OD), Figure S26: DQF-COSY spectrum of **3** (600 MHz, CD_3_OD), Figure S27: HSQC spectrum of **3** (600 MHz, CD_3_OD), Figure S28: HMBC spectrum of **3** (600 MHz, CD_3_OD), Figure S29: IR spectrum of **3**, Figure S30: ^1^H NMR spectrum of **4** (400 MHz, CD_3_OD), Figure S31: ^1^H NMR spectrum of **5** (400 MHz, CD_3_OD), Figure S32: ^1^H NMR spectrum of **6** (400 MHz, CD_3_OD), Figure S33: Anti-oomycete activity of compounds **1**–**6**.
