# Supplementary material for: New Crambescidin-Type Alkaloids from the Indonesian Marine Sponge Clathria bulbotoxa
Source: Mar Drugs. 2018 Mar 8;16(3):84. doi: 10.3390/md16030084 (PMC5867628; doi:10.3390/md16030084)
Supplement: Supplementary file 1 [file marinedrugs-16-00084-s001.pdf]

Supplementary Materials

New Crambescidin-type Alkaloids from the Indonesian Marine Sponge *Clathria bulbotoxa*

Kasmiati Kasmiati[1,2], Yukio Yoshioka[3], Tetsuji Okamoto[3], and Makoto Ojika[1,*]

Contents

Table S1: NMR data of **2** (600 MHz, acetone-d_6)

Figure S1: ^{1}H NMR spectrum of **1** (600 MHz, CD_3OD)

Figure S2: ^{1}H NMR spectrum of **1** (400 MHz, $CDCl_3$)

Figure S3: ^{13}CNMR spectrum of **1** (150 MHz, CD_3OD)

Figure S4: DQF-COSY spectrum of **1** (600 MHz, CD_3OD)

Figure S5: HSQC spectrum of **1** (600 MHz, CD_3OD)

Figure S6: HMBC spectrum of **1** (600 MHz, CD_3OD)

Figure S7: NOESY spectrum of **1** (600 MHz, CD_3OD)

Figure S8: IR spectrum of **1**

Figure S9: ESI-TOF-MS(+) spectra of **1**–**3**

Figure S10: ^{1}H NMR spectrum of **2** (400 MHz, CD_3OD)

Figure S11: ^{13}CNMR spectrum of **2** (100 MHz, CD_3OD)

Figure S12: DQF-COSY spectrum of **2** (400 MHz, CD_3OD)

Figure S13: HSQC spectrum of **2** (400 MHz, CD_3OD)

Figure S14: HMBC spectrum of **2** (400 MHz, CD_3OD)

Figure S15: NOESY spectrum of **2** (400 MHz, CD_3OD)

Figure S16: IR spectrum of **2**

Figure S17: ^{1}H NMR spectrum of **2** (600 MHz, acetone-d_6)

Figure S18: ^{13}C NMR spectrum of **2** (150 MHz, acetone-d_6)

Figure S19: DQF-COSY spectrum of **2** (600 MHz, acetone-d_6)

Figure S20: HSQC spectrum of **2** (600 MHz, acetone-d_6)

Figure S21: HMBC spectrum of **2** (600 MHz, acetone-d_6)

Figure S22: NOESY spectrum of **2** (600 MHz, acetone-d_6)

Figure S23: ^{1}H NMR spectrum of **3** (600 MHz, CD_3OD)

Figure S24: ^{1}H NMR spectrum of **3** (400 MHz, $CDCl_3$)

Figure S25: ^{13}C NMR spectrum of **3** (150 MHz, CD_3OD)

Figure S26: DQF-COSY spectrum of **3** (600 MHz, CD_3OD)

Figure S27: HSQC spectrum of **3** (600 MHz, CD_3OD)

Figure S28: HMBC spectrum of **3** (600 MHz, CD_3OD)

Figure S29: IR spectrum of **3**

Figure S30: ^{1}H NMR spectrum of **4** (400 MHz, CD_3OD)

Figure S31: ^{1}H NMR spectrum of **5** (400 MHz, CD_3OD)

Figure S32: ^{1}H NMR spectrum of **6** (400 MHz, CD_3OD)

Figure S33: Anti-oomycete activity of compounds **1**–**6**

Table S1: NMR data of **2** (600 MHz, acetone-d_6)

Positition	δC (ppm)	δH (ppm), m, *J* (Hz)
1	13.7, CH_3	0.85, t (7.2)
2	19.1, CH_2	1.34, m
3	38.4, CH_2	1.37, m
4	69.9, CH	3.70, t (9.9)
5a	31.6, CH_2	1.19, m
5b		1.59, m
6a	18.9, CH_2[a]	1.62, m
6b		2.29, m
7a	34.4, CH_2[b]	1.62, m
7b		1.74, m
8	81.0, C[c]	
9a	40.3, CH_2[d]	1.62, m
9b		2.16, t (12.9)[g]
10	53.2, CH[e]	4.00, m
11a	30.5, CH_2[f]	1.78, m
11b		2.31, m
12a	30.4, CH_2[f]	1.78, m
12b		2.31, m
13	52.7, CH[e]	4.00, m
14a	40.4, CH_2[d]	1.62, m
14b		2.15, t (12.9)[g]
15	81.1, C[c]	
16a	34.3, CH_2[b]	1.62, m
16b		1.74, m
17a	18.7, CH_2[a]	1.62, m
17b		2.29, m
18a	33.1, CH_2	1.16, m
18b		1.64, m
19	67.1, CH	3.80, m
20	22.1, CH_3	1.06, d (6.0)
21	149.4, C	
NHa		10.13, s
NHb		10.16, s

The number of hydrogen on carbon was determined by HSQC. [a-g] Interchangeable signals within the same marks.

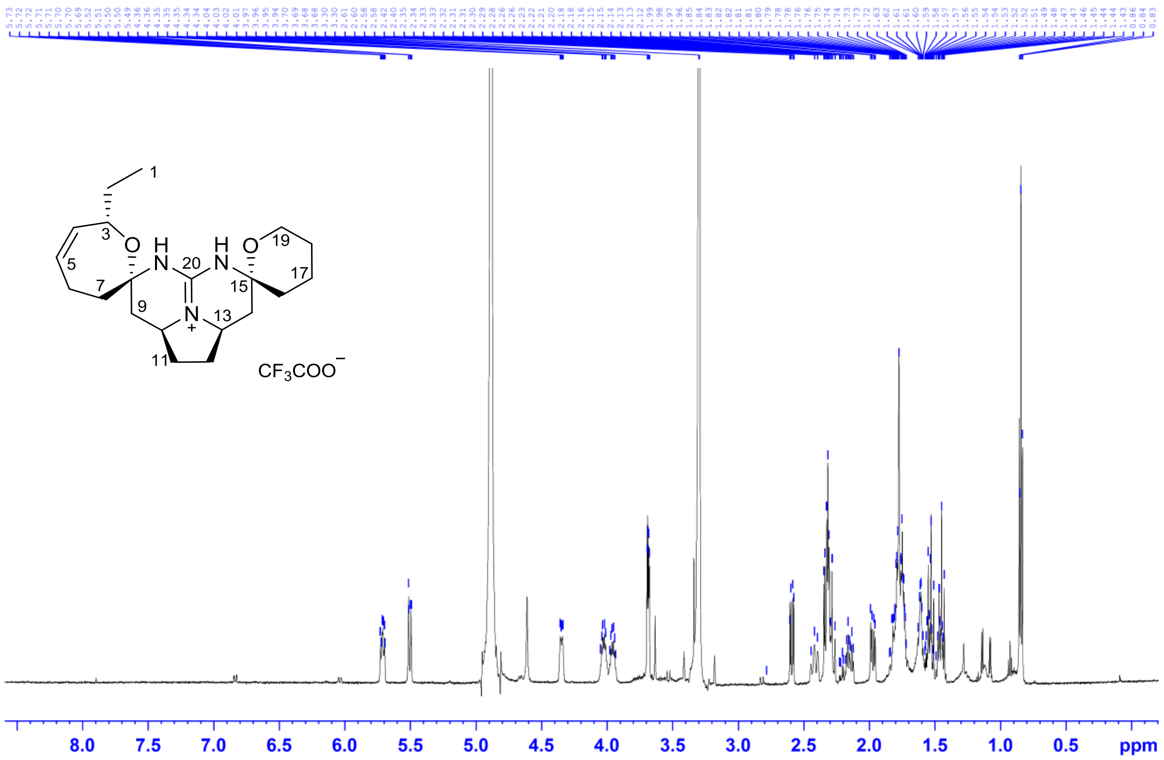

Figure S1: ^{1}H NMR spectrum of **1** (600 MHz, CD_3OD).

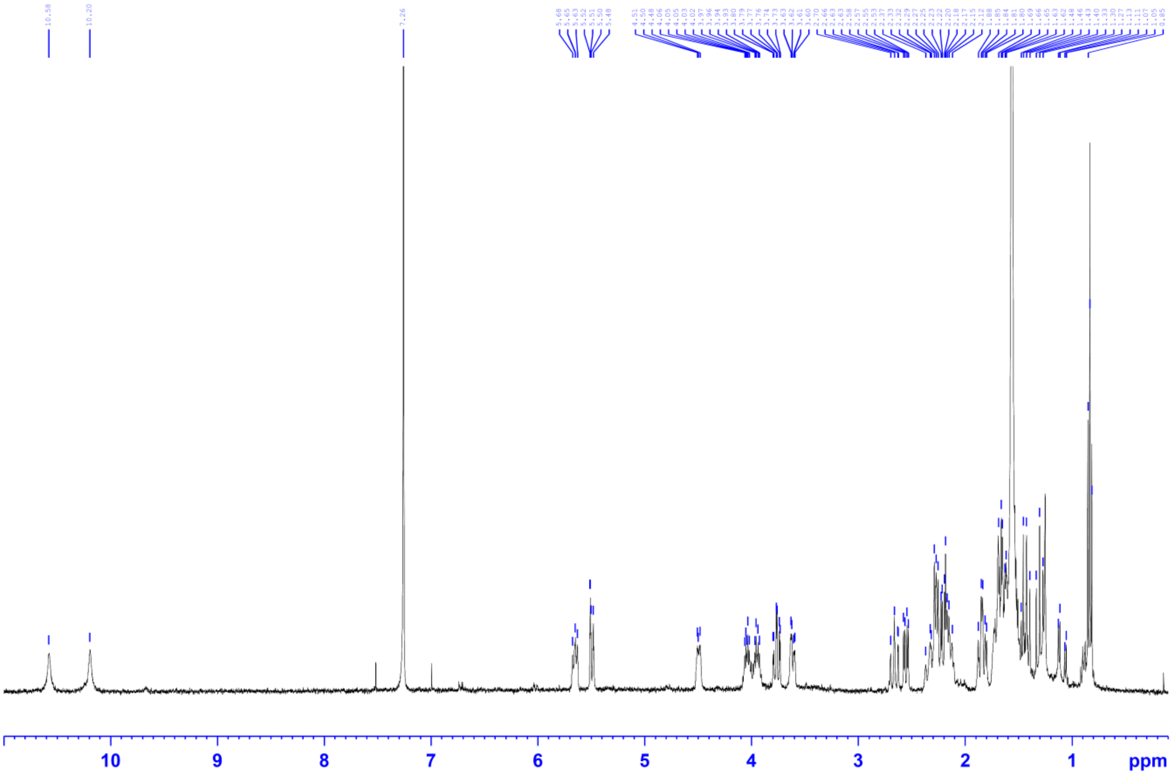

Figure S2: ^{1}H NMR spectrum of **1** (400 MHz, $CDCl_3$).

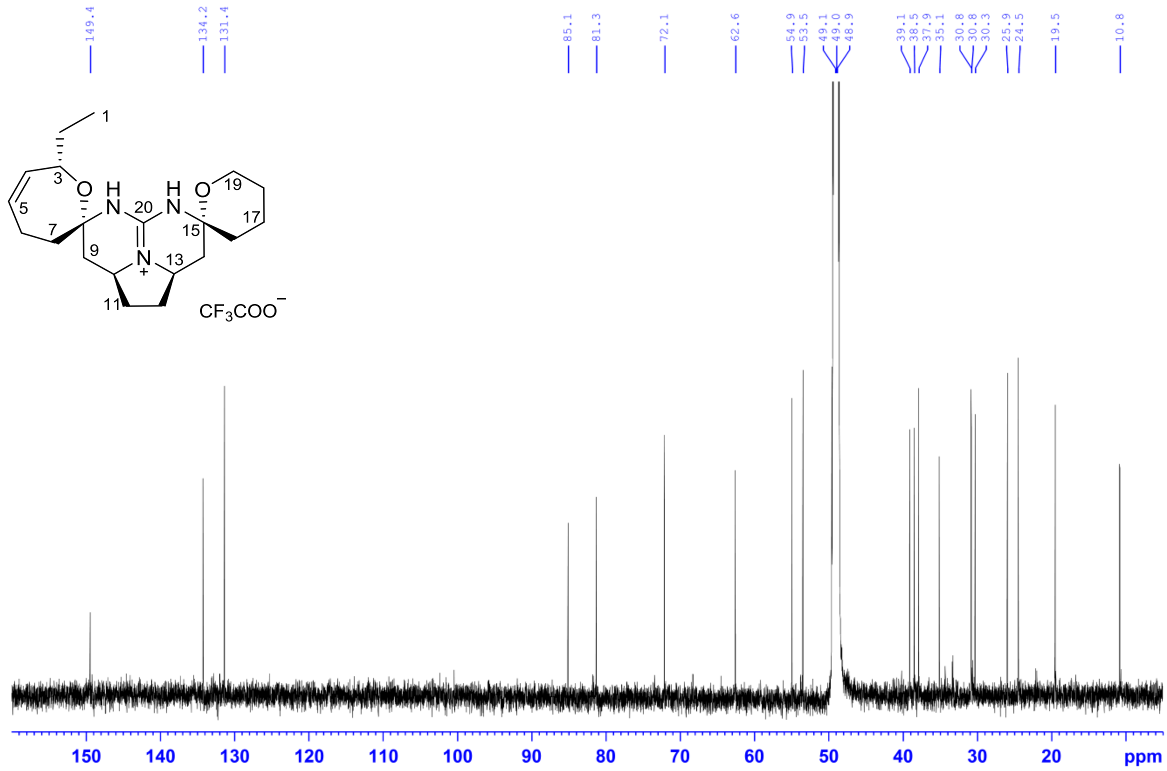

Figure S3: ^{13}C NMR spectrum of **1** (150 MHz, CD_3OD).

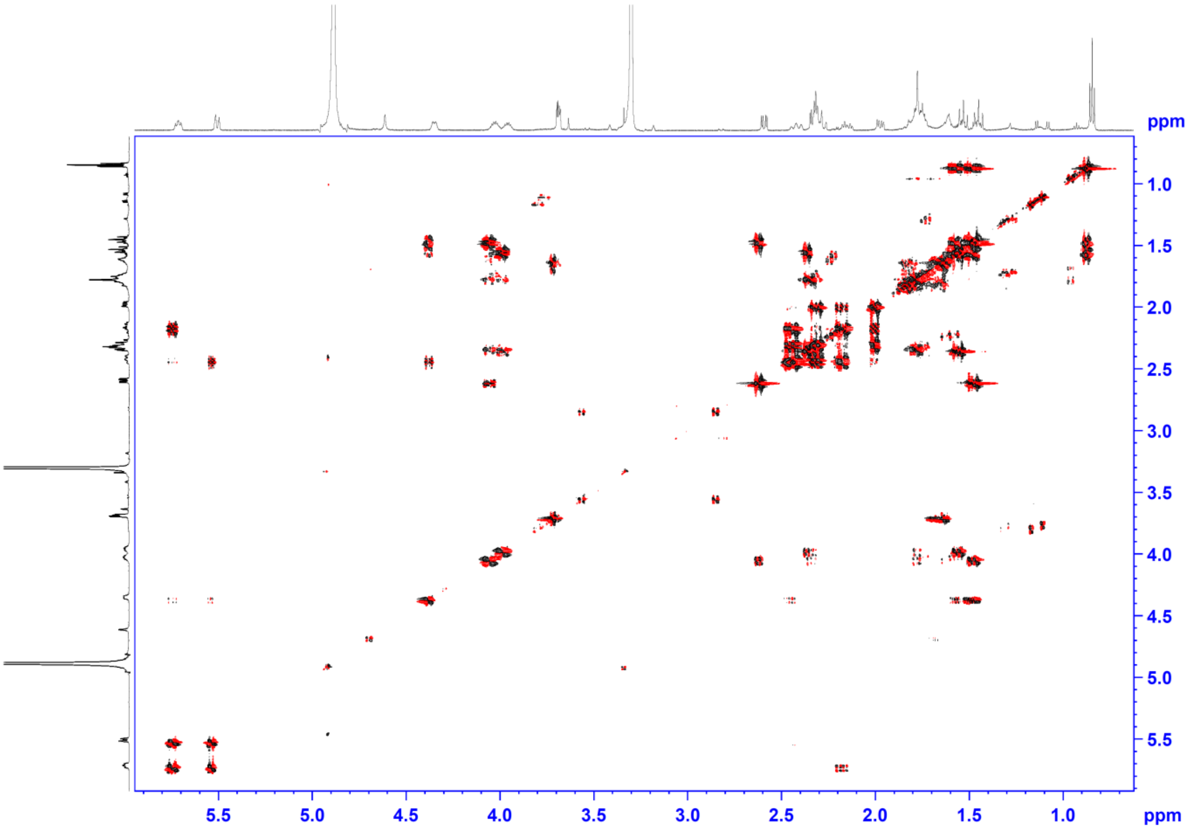

Figure S4: DQF-COSY spectrum of **1** (600 MHz, CD_3OD).

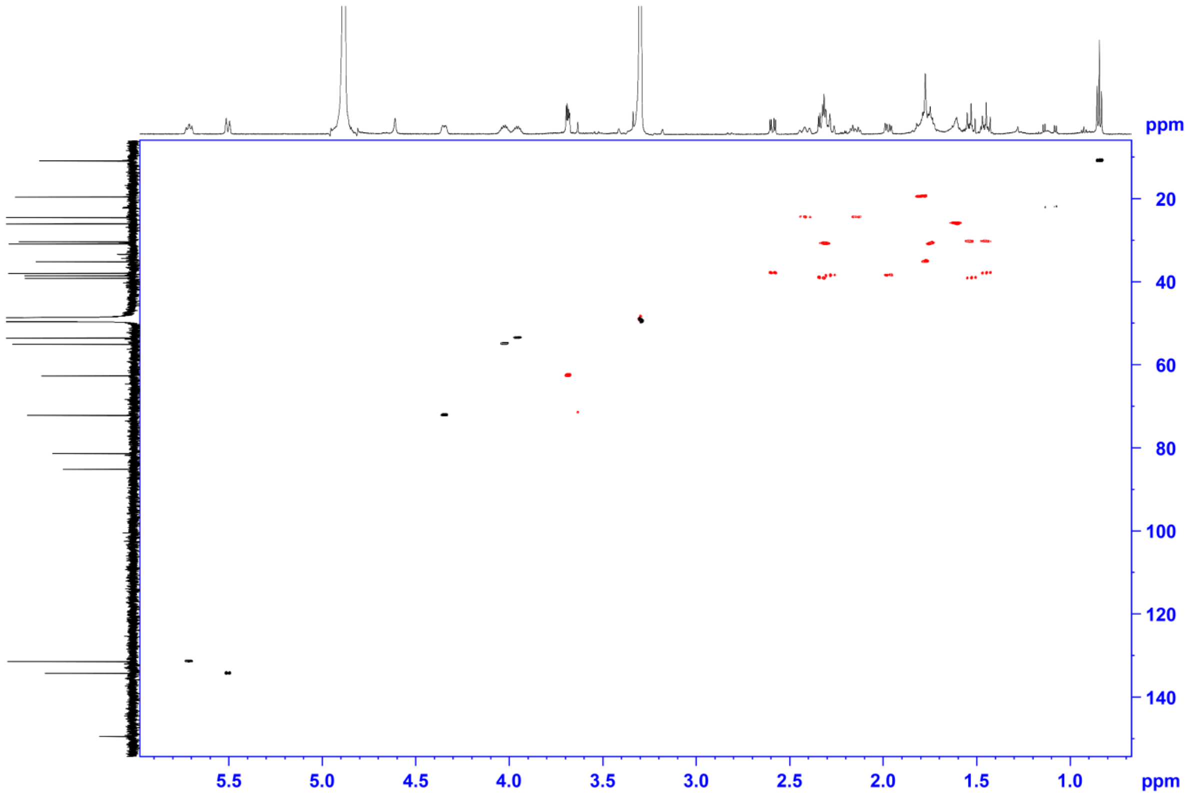

Figure S5: HSQC spectrum of **1** (600 MHz, CD_3OD).

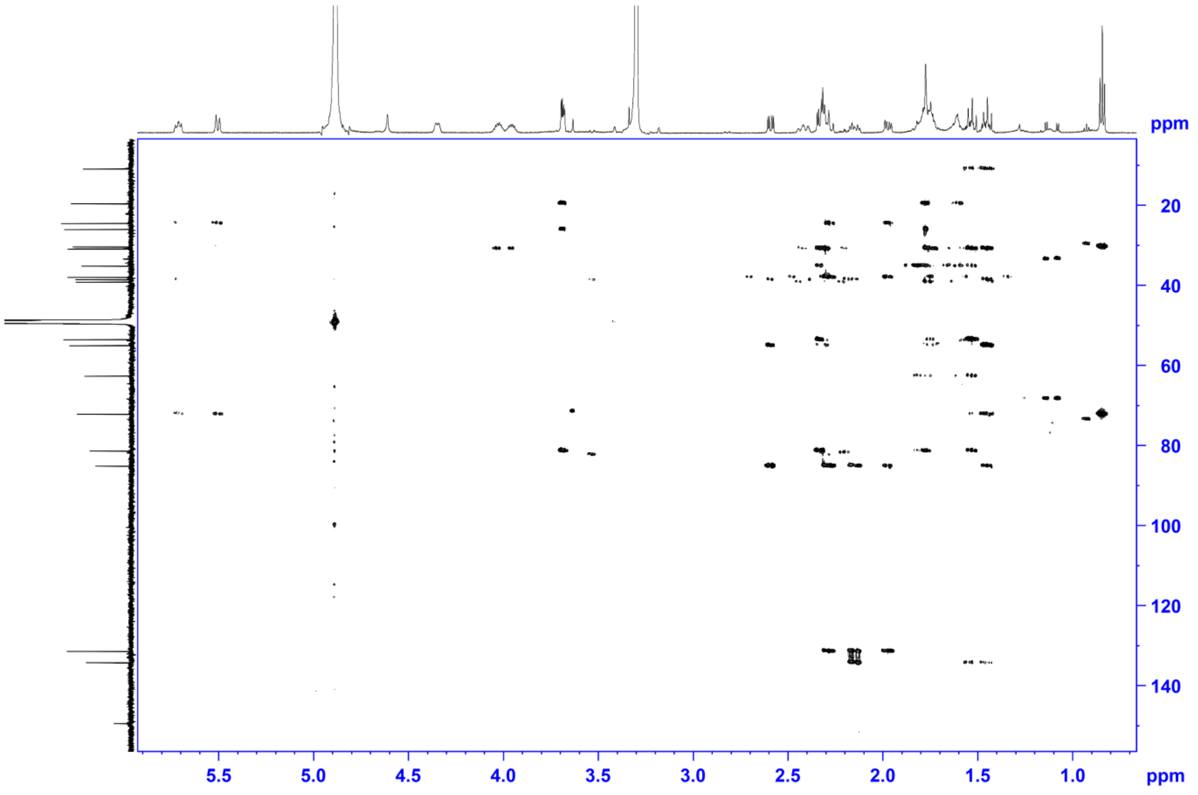

Figure S6: HMBC spectrum of **1** (600 MHz, CD_3OD).

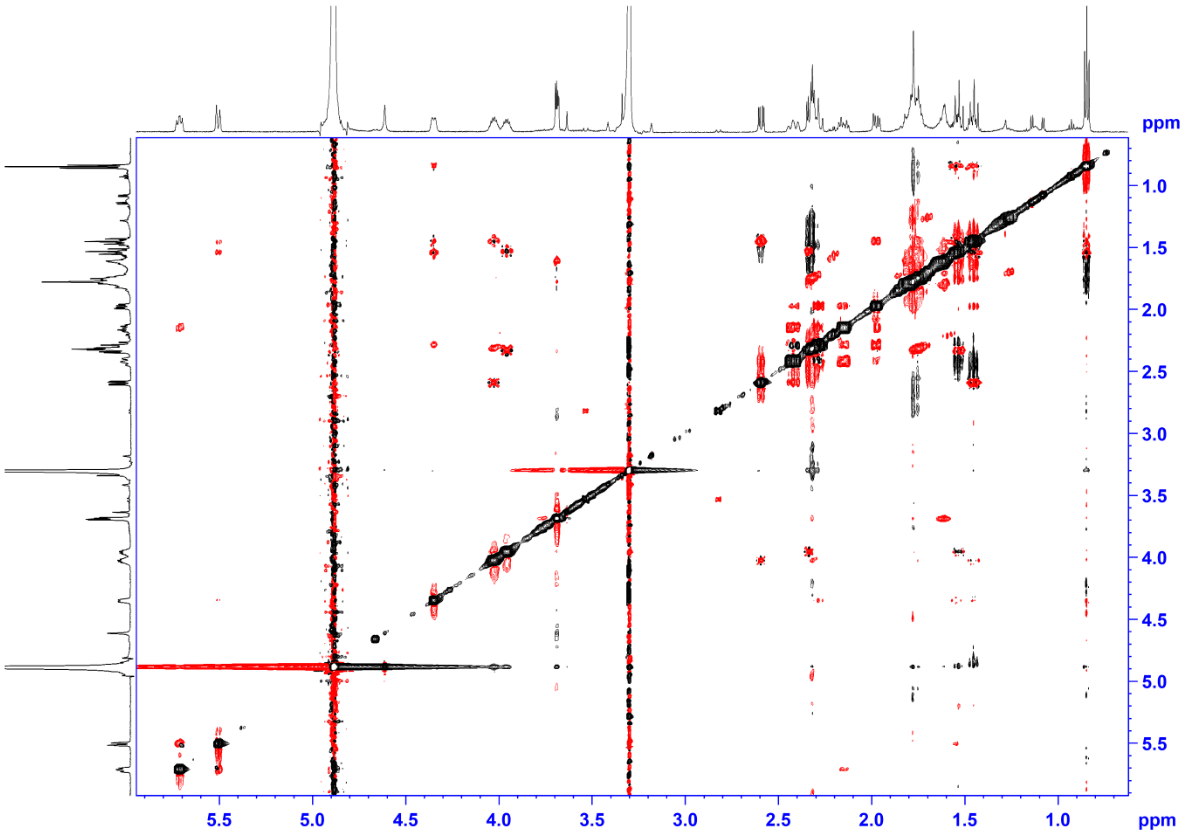

Figure S7: NOESY spectrum **1** (600 MHz, CD_3OD).

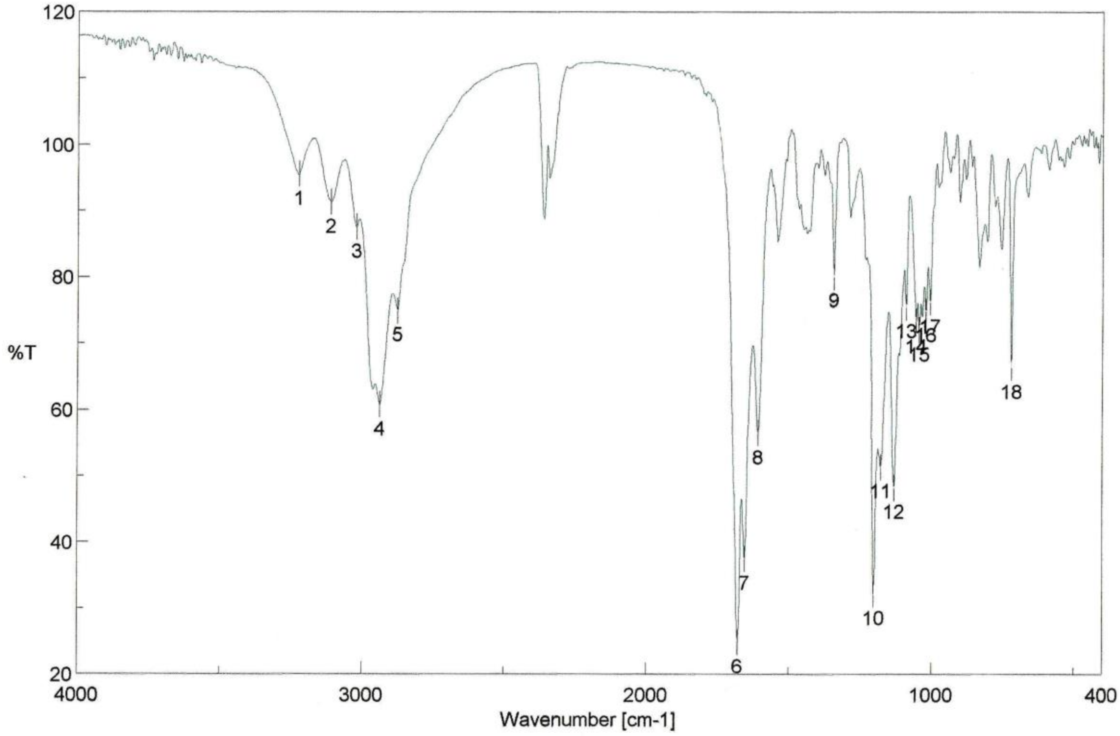

Figure S8: IR spectrum of **1.**

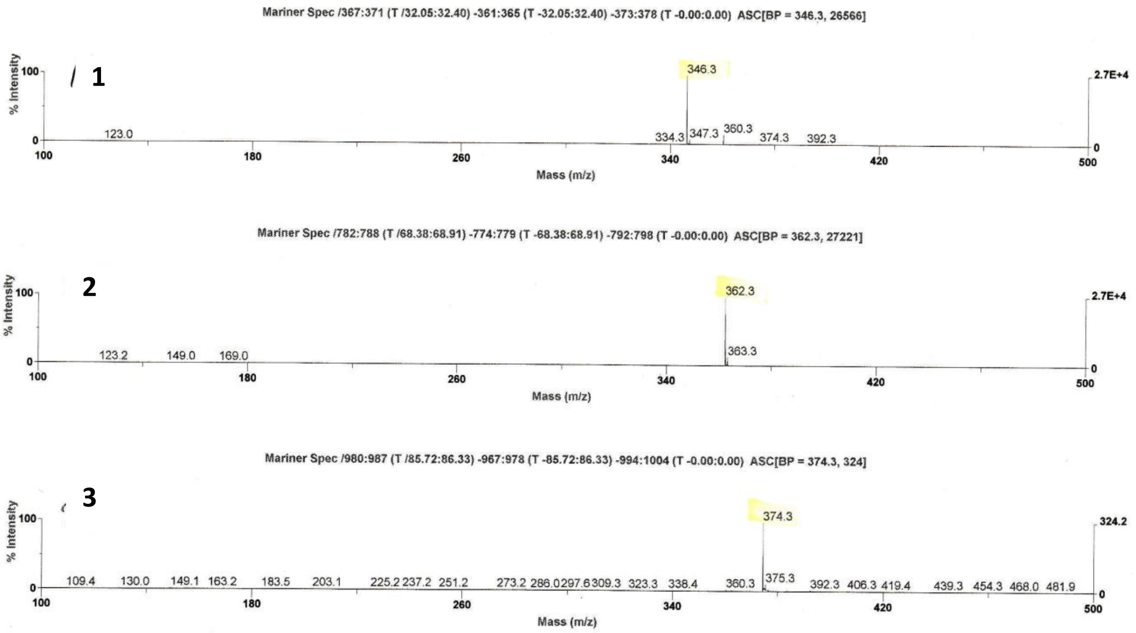

Figure S9: ESI-TOF-MS(+) spectra of **1**–**3.**

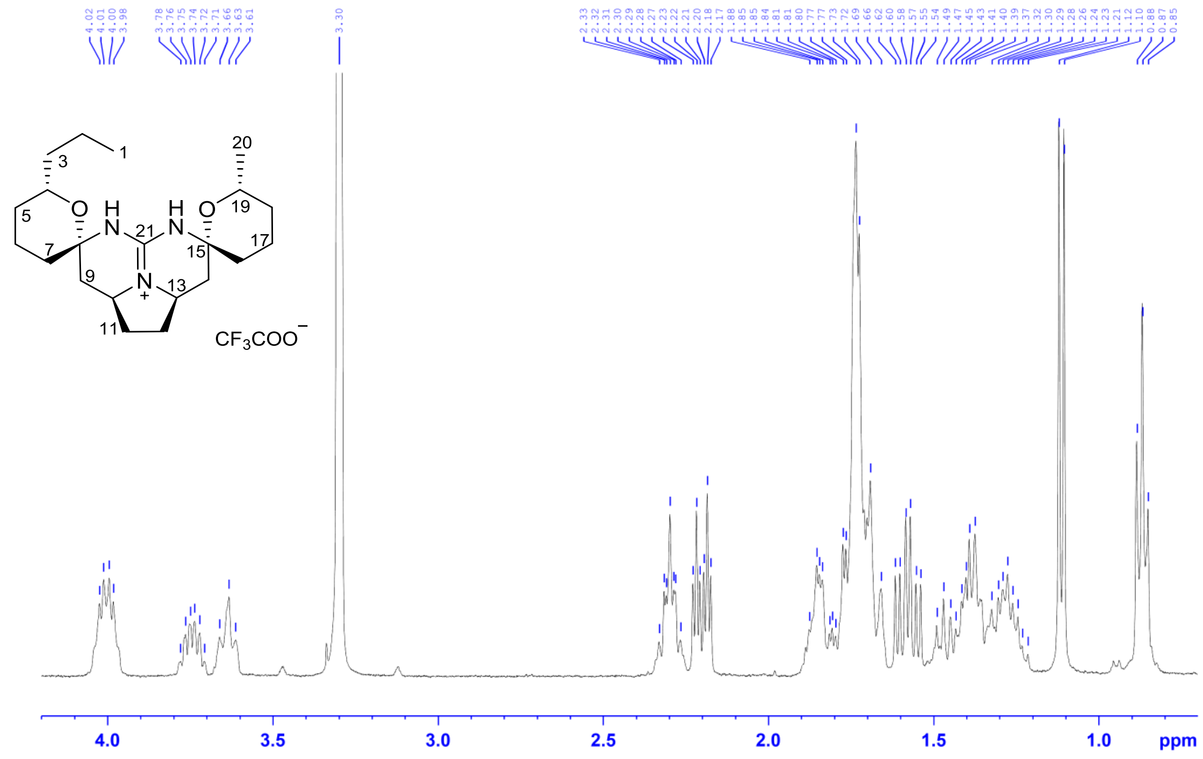

Figure S10: ^{1}H NMR spectrum of **2** (400 MHz, CD3OD).

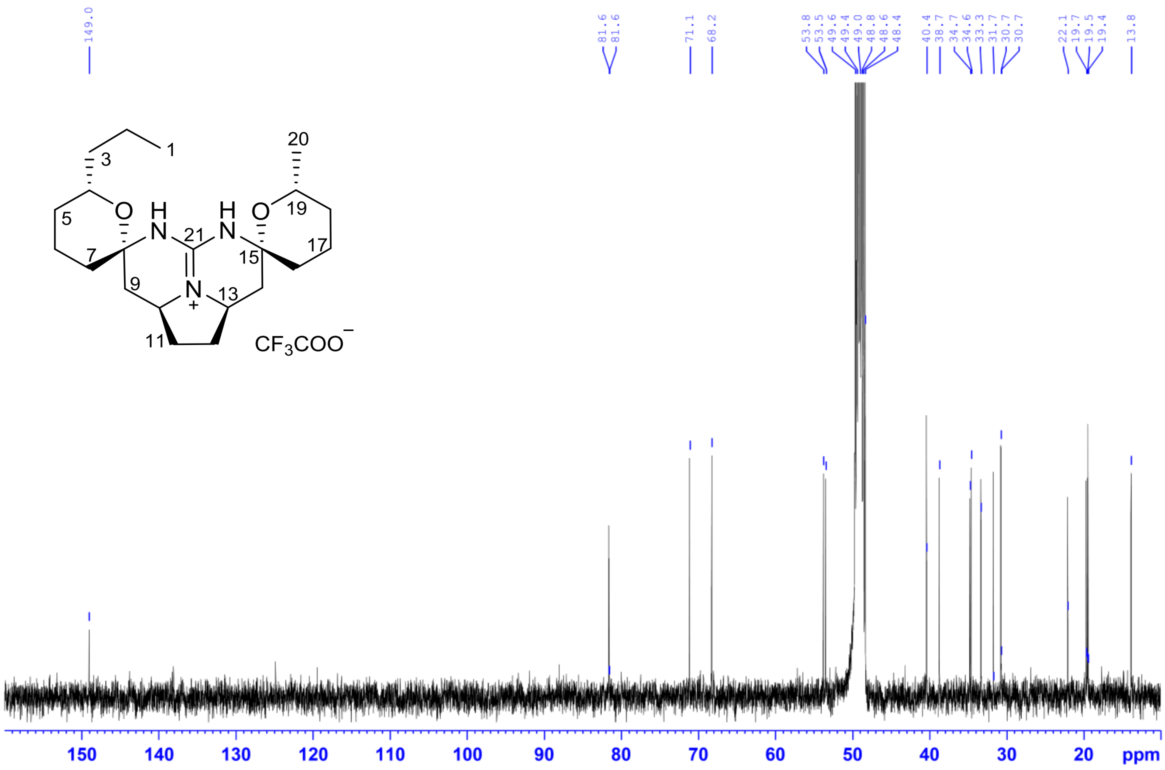

Figure S11: ^{13}C NMR spectrum of **2** (100 MHz, CD_3OD).

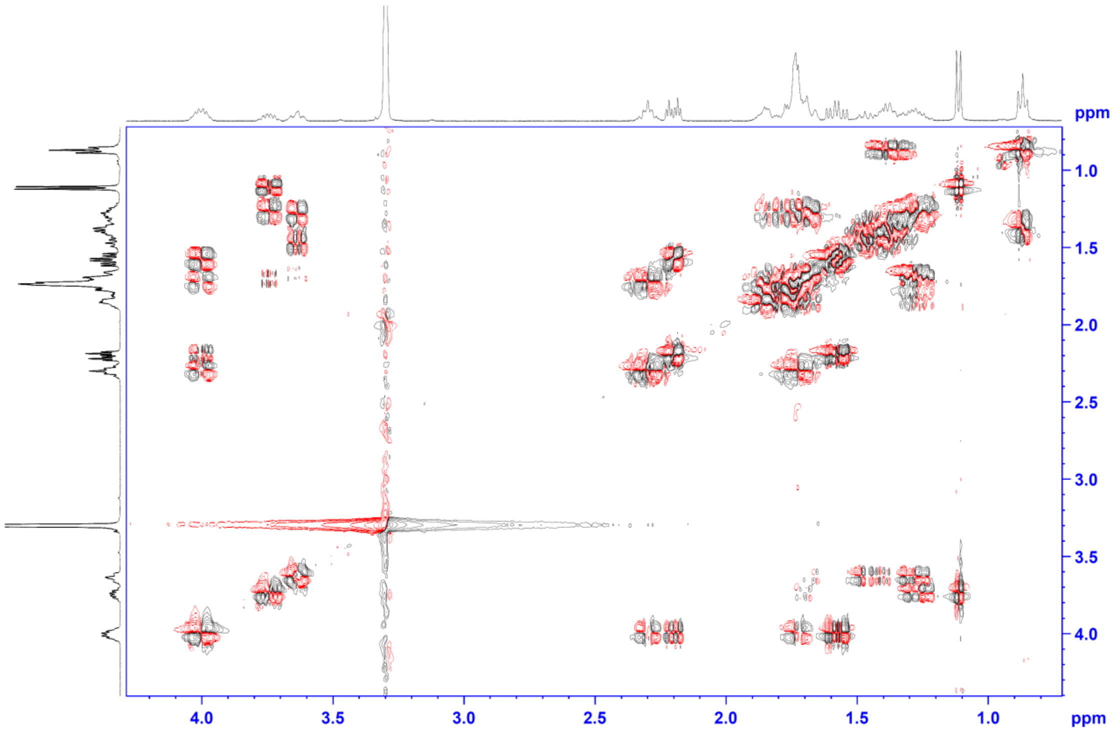

Figure S12: DQF-COSY spectrum of **2** (400 MHz, CD_3OD).

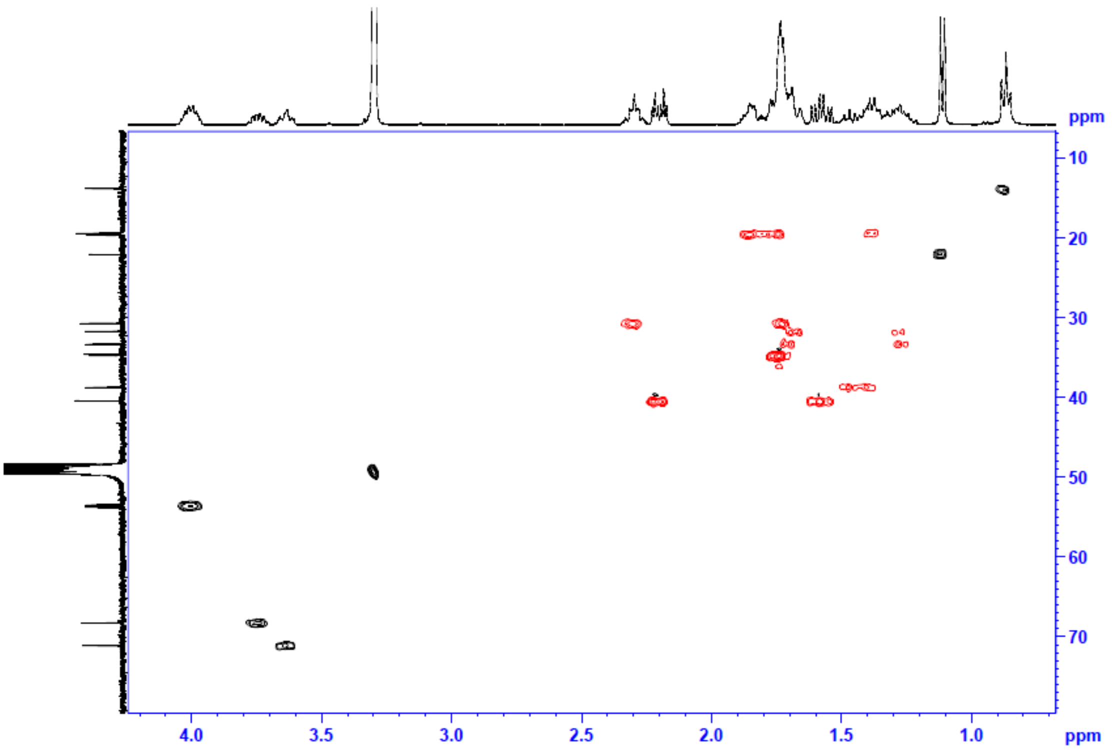

Figure S13: HSQC spectrum of **2** (400 MHz, CD_3OD).

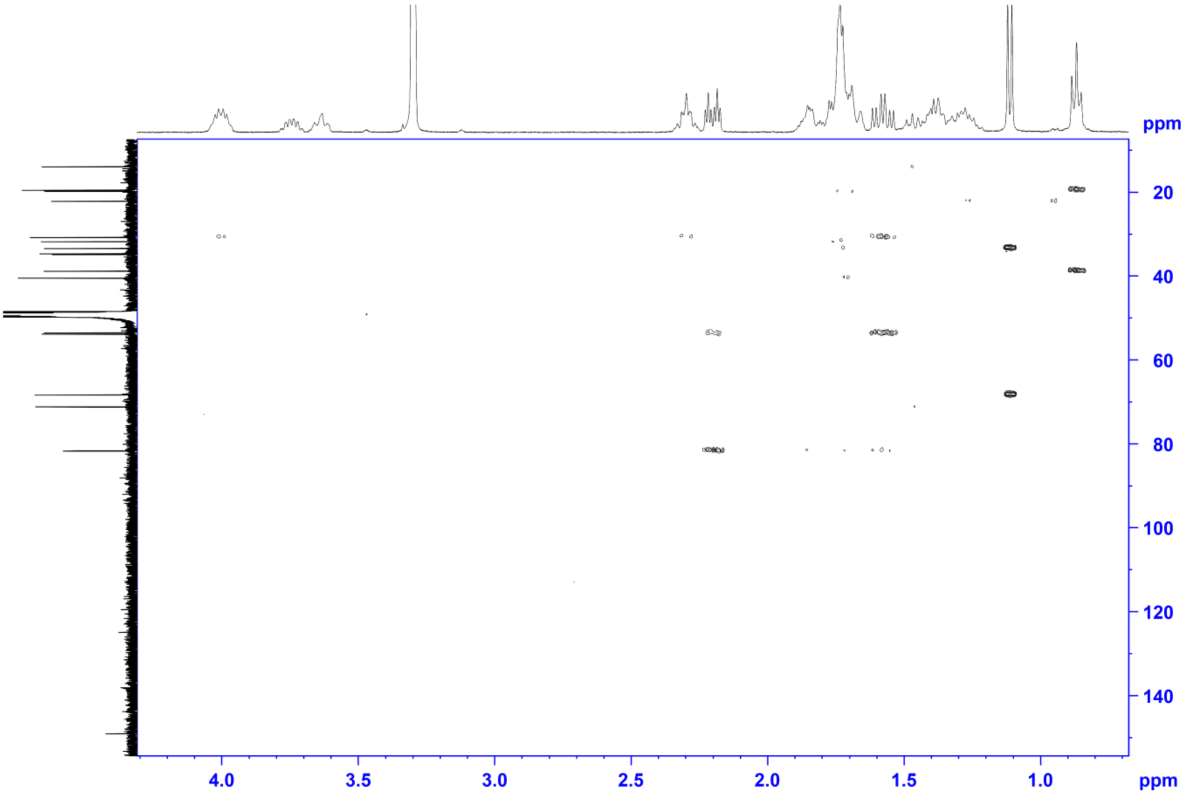

Figure S14: HMBC spectrum of **2** (400 MHz, CD_3OD).

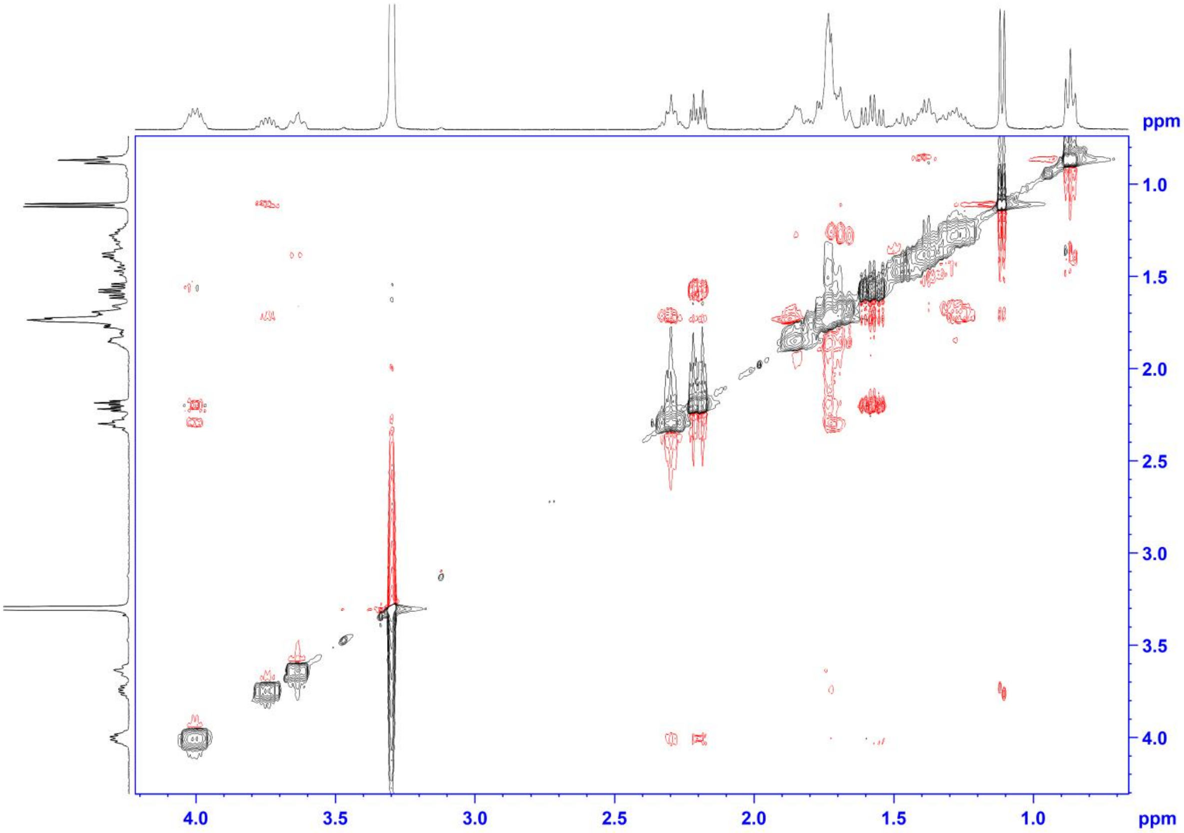

Figure S15: NOESY spectrum of **2** (400 MHz, CD_3OD).

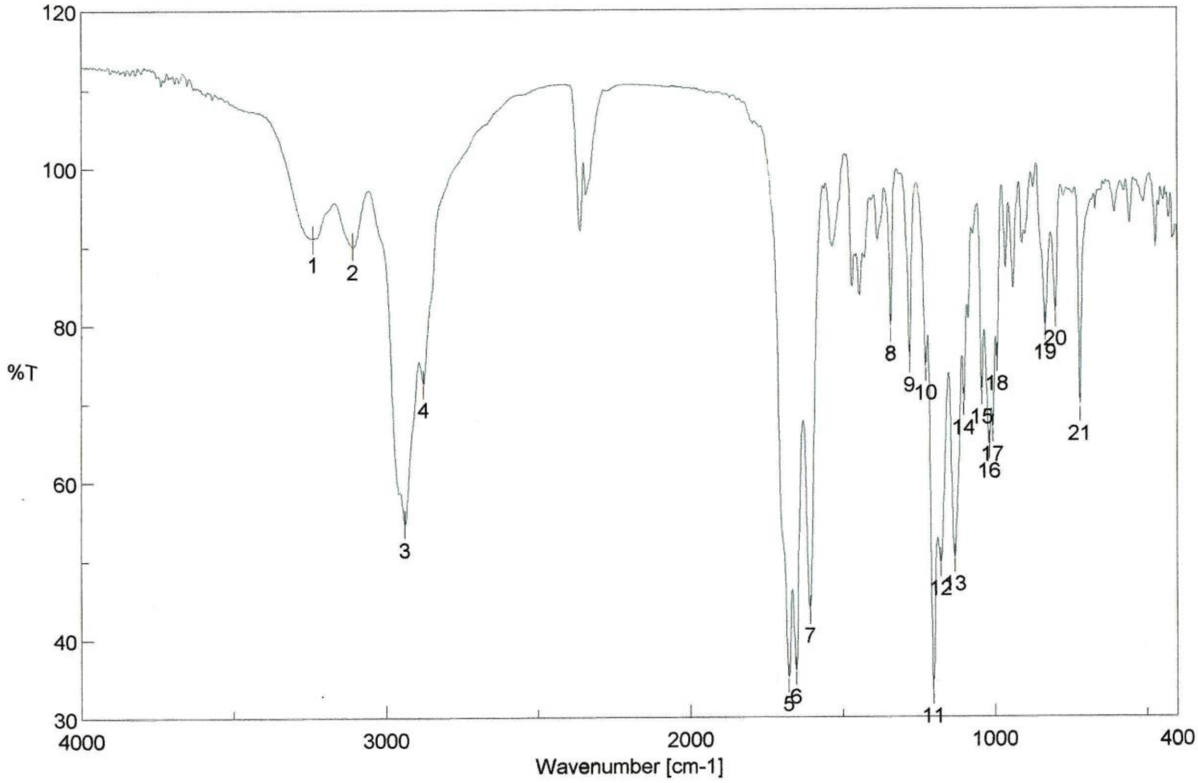

Figure S16: IR spectrum of **2.**

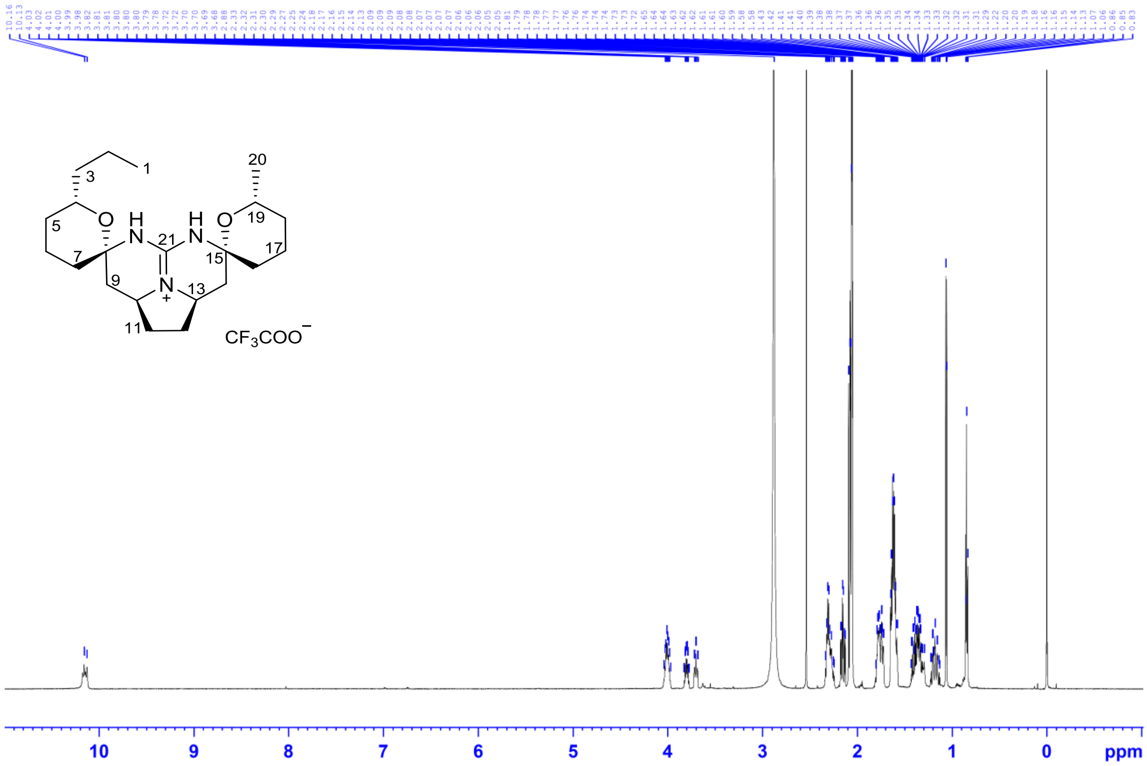

Figure S17: ^{1}H NMR spectrum of **2** (600 MHz, acetone-*d*6).

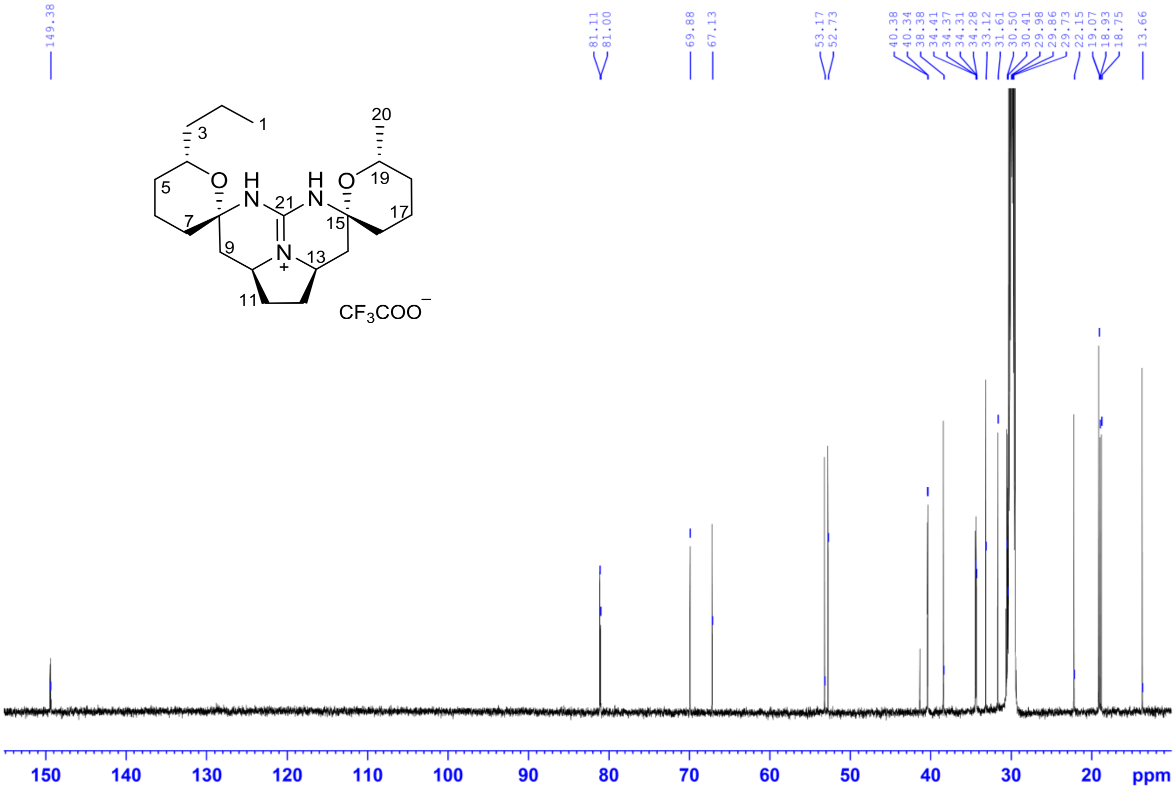

Figure S18: ^{13}C NMR spectrum of **2** (150 MHz, acetone-*d*6).

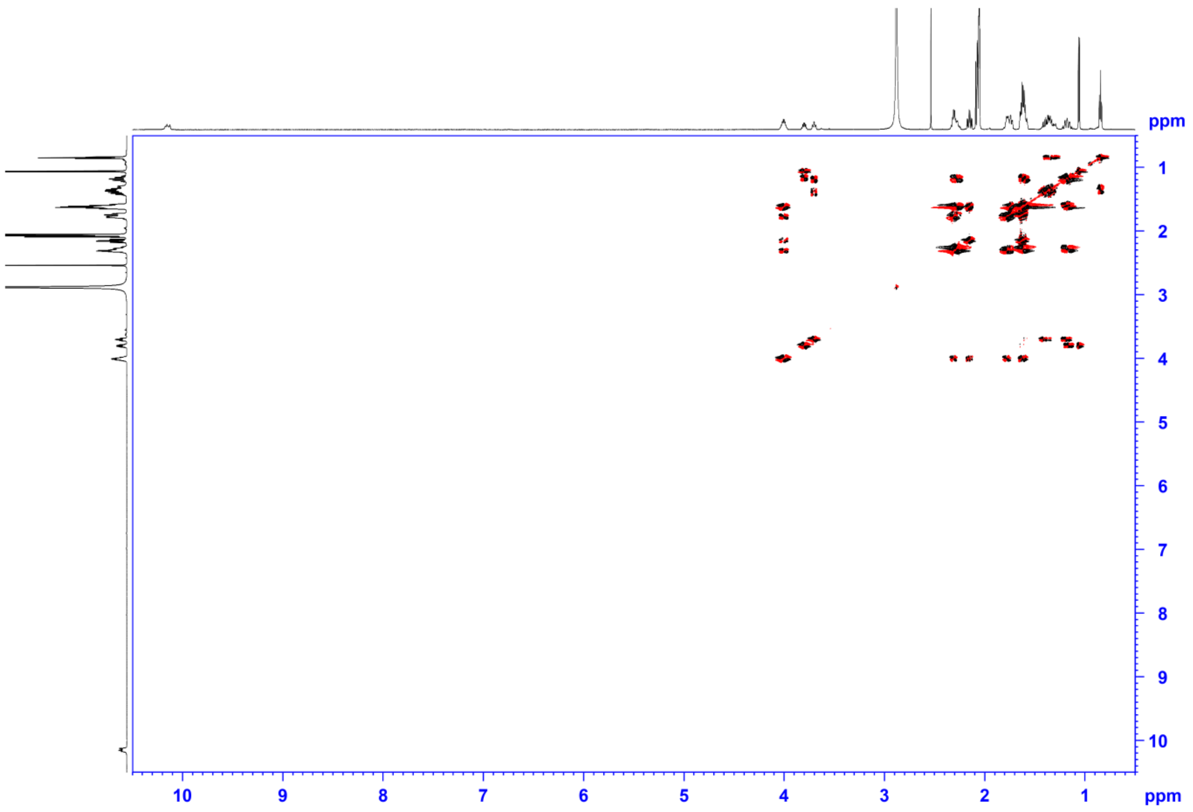

Figure S19: DQF-COSY spectrum of **2** (600 MHz, acetone-d_6).

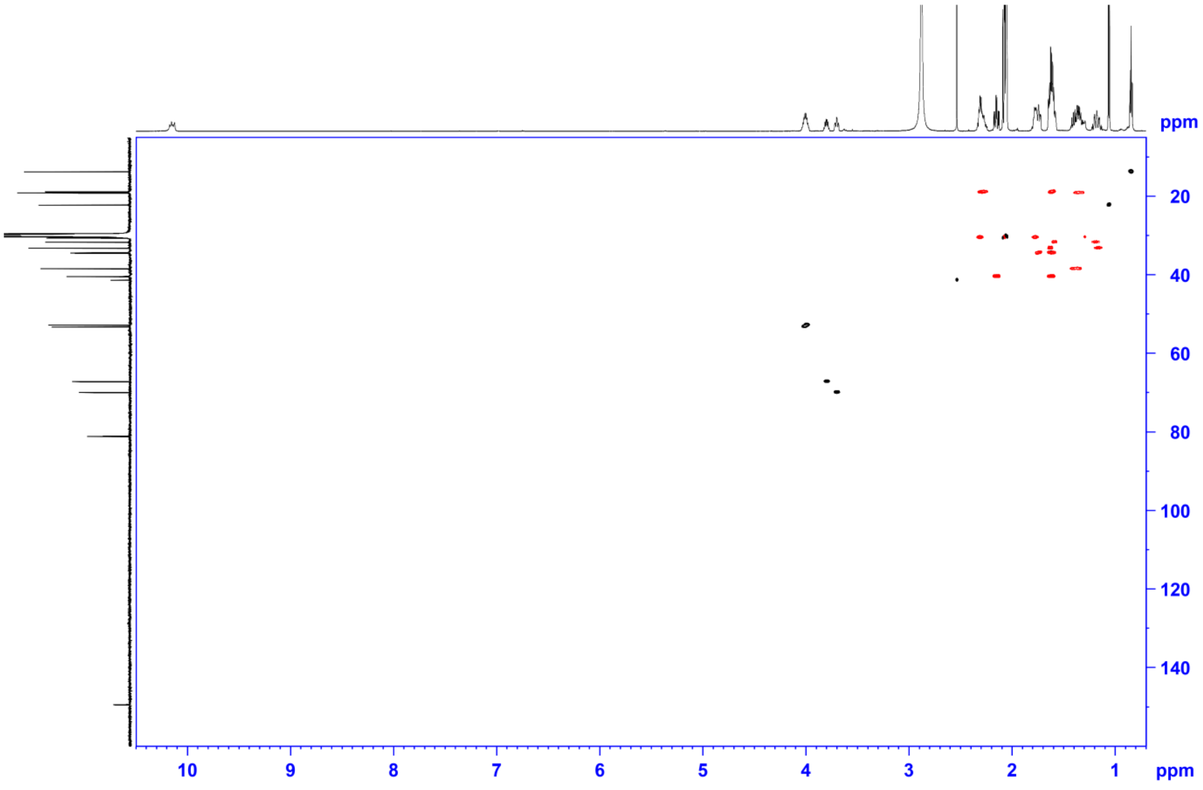

Figure S20: HSQC spectrum of **2** (600 MHz, acetone-d_6).

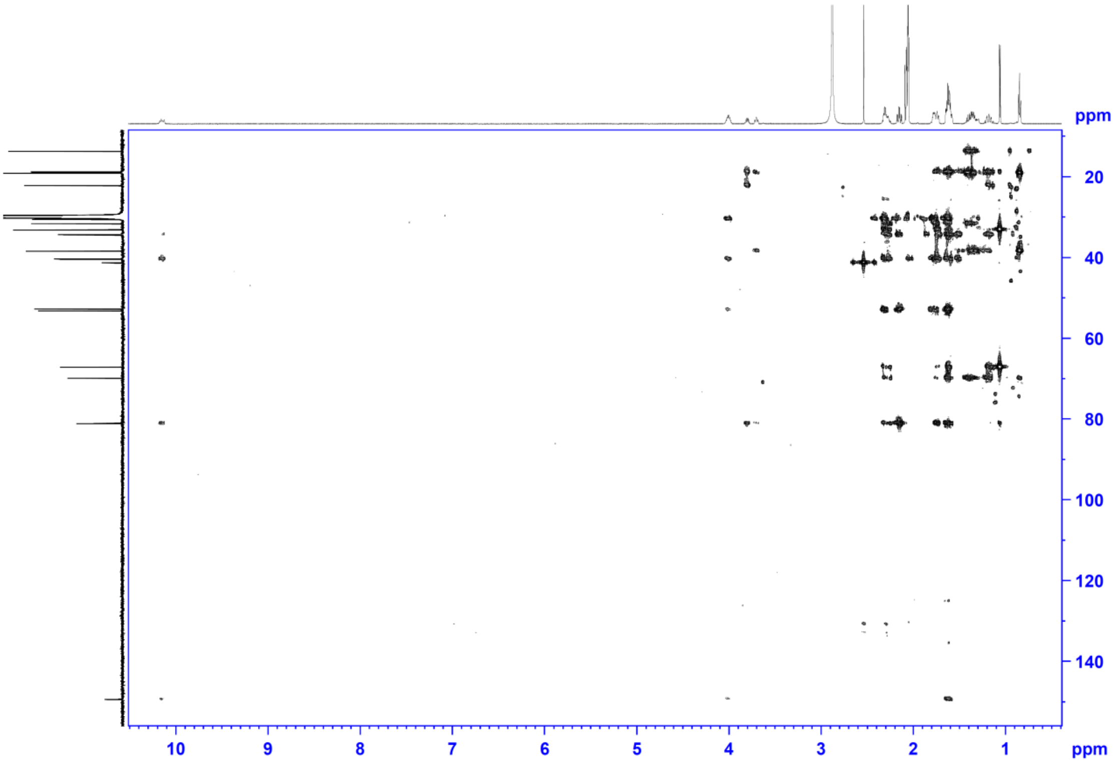

Figure S21: HMBC spectrum of **2** (600 MHz, acetone-d_6).

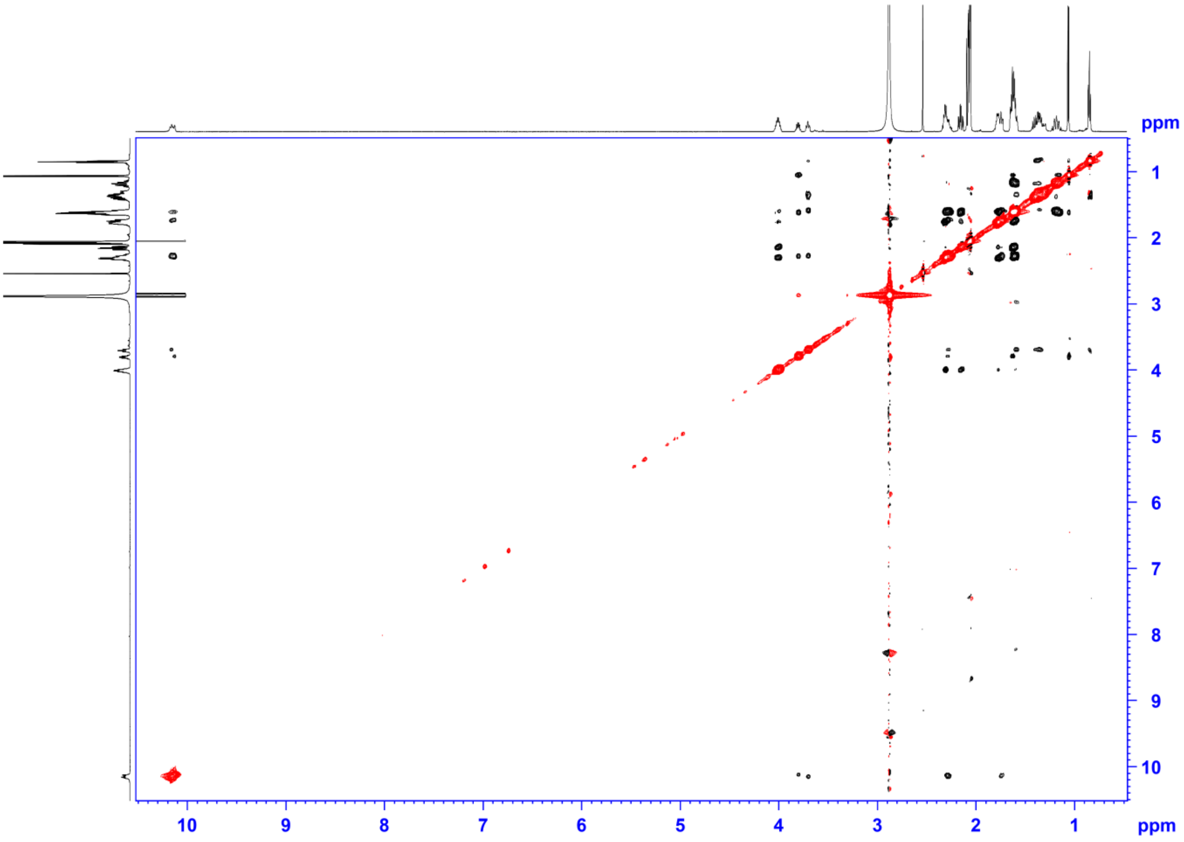

Figure S22: NOESY spectrum of **2** (600 MHz, acetone-d_6).

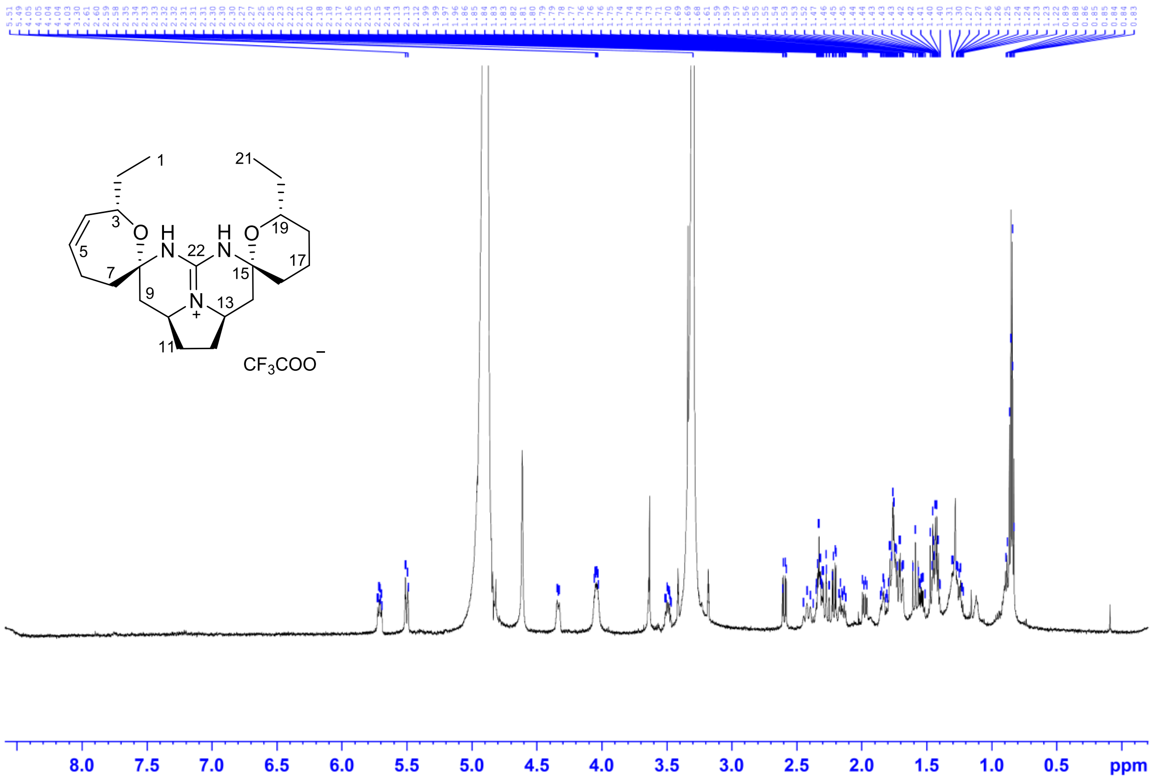

Figure S23: ¹H NMR spectrum of **3** (600 MHz, CD_3OD).

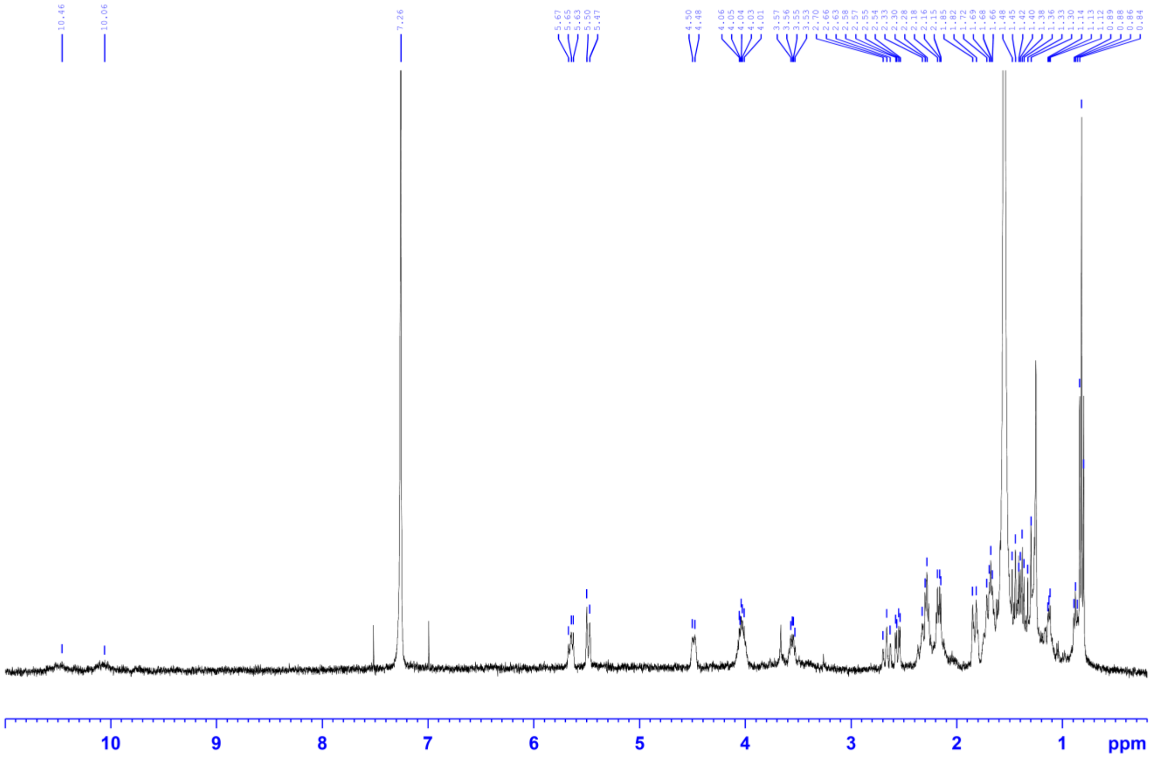

Figure S24: ¹H NMR spectrum of **3** (400 MHz, $CDCl_3$).

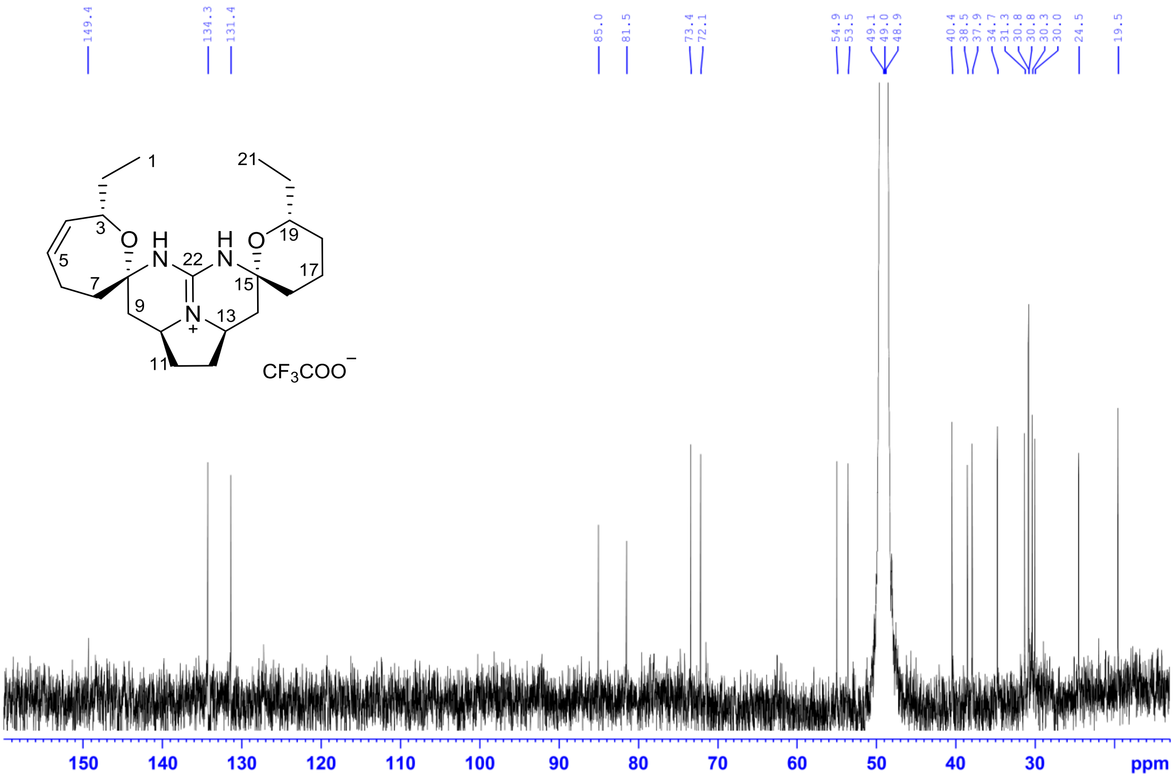

Figure S25: ^{13}C NMR spectrum of **3** (150 MHz, CD_3OD).

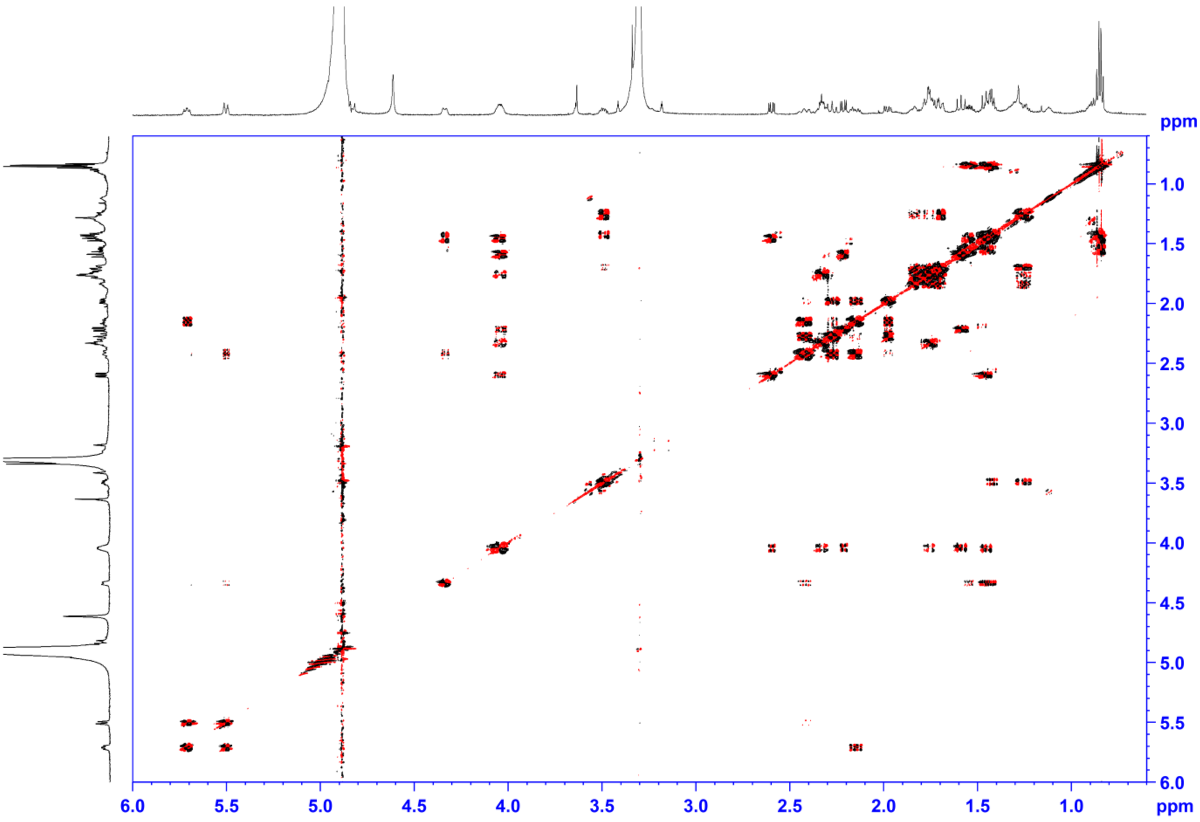

Figure S26: DQF-COSY spectrum of **3** (600 MHz, CD_3OD).

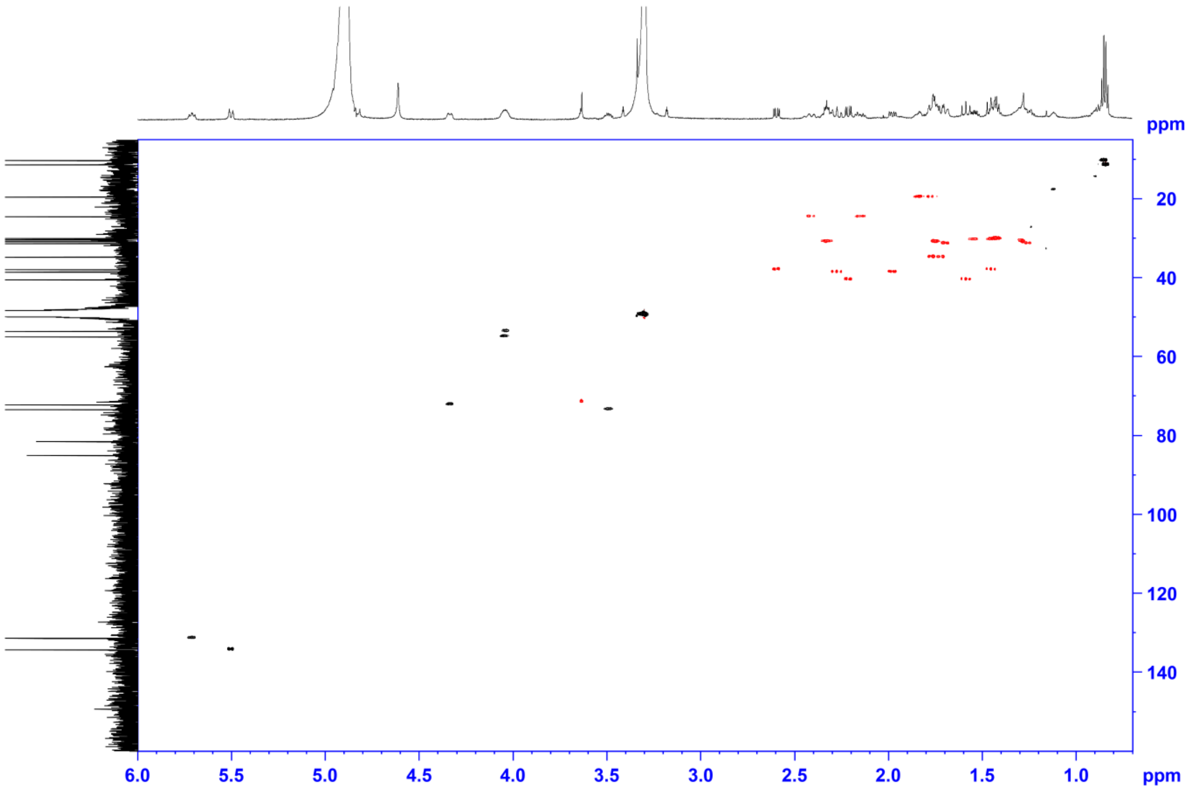

Figure S27: HSQC spectrum of **3** (600 MHz, CD_3OD).

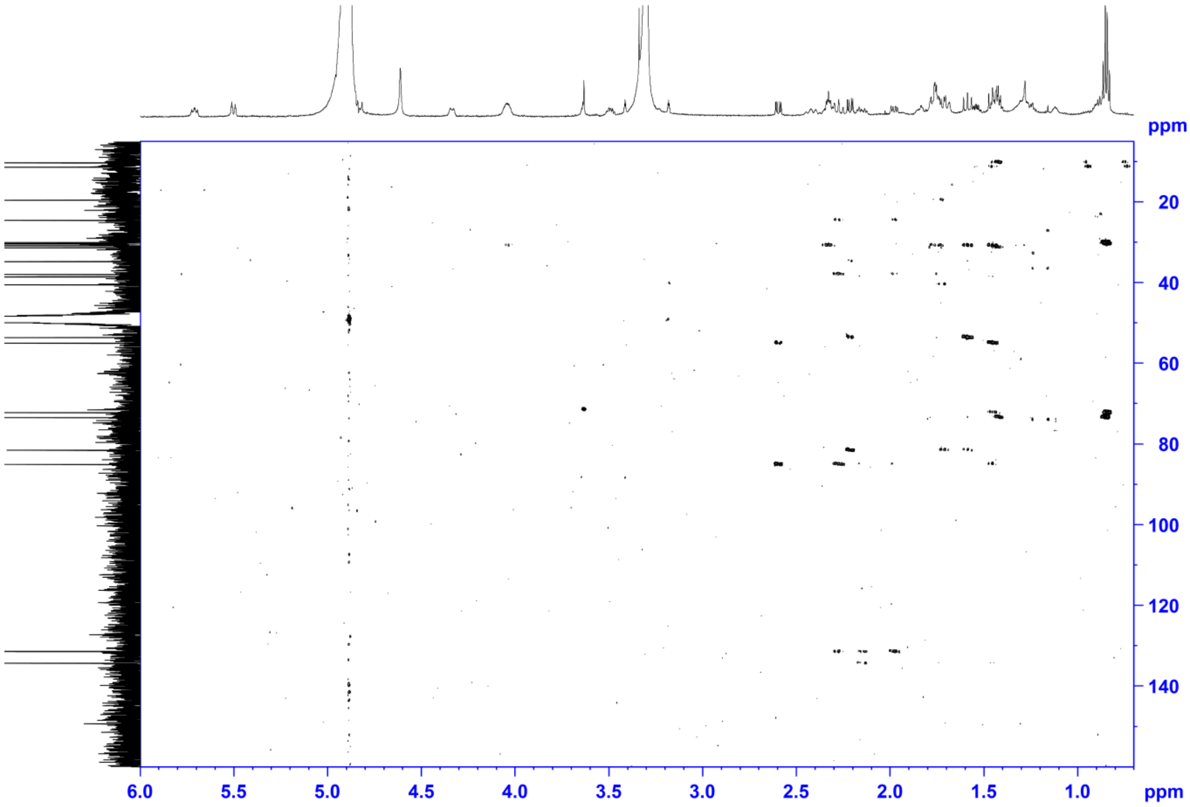

Figure S28: HMBC spectrum **3** (600 MHz, CD_3OD).

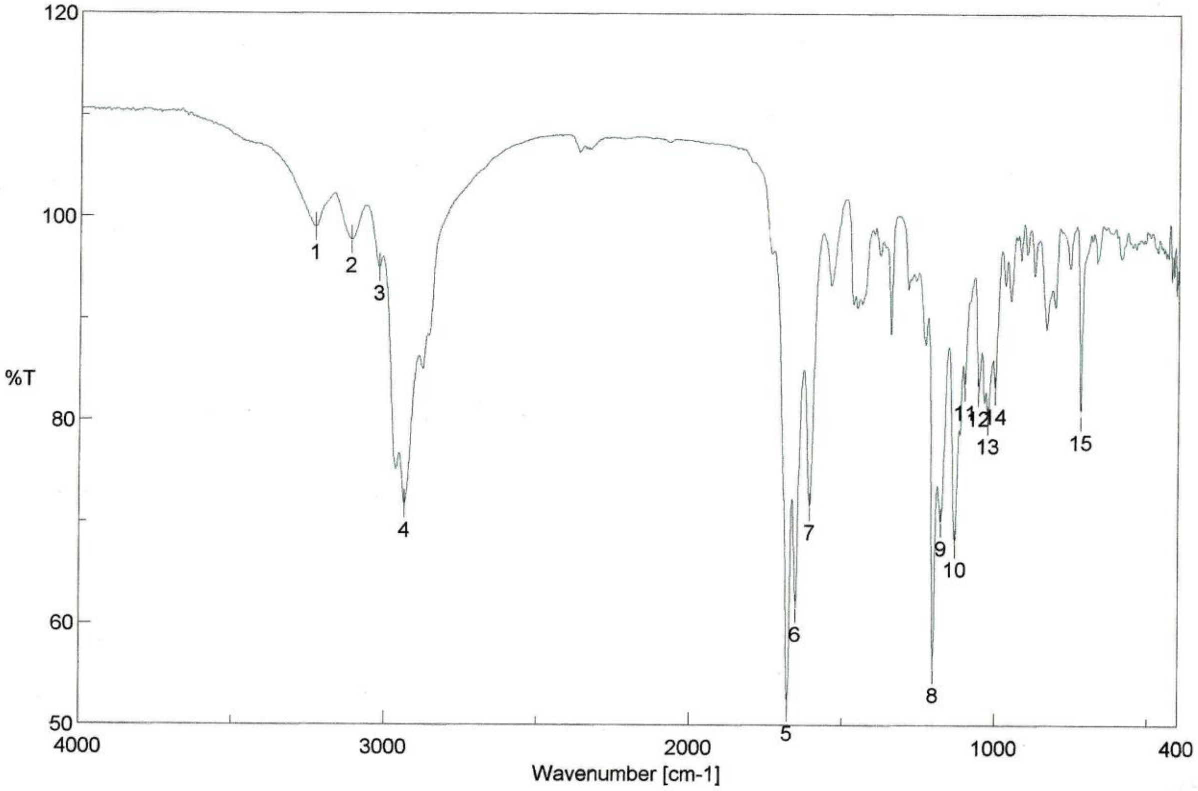

Figure S29: IR spectrum of **3.**

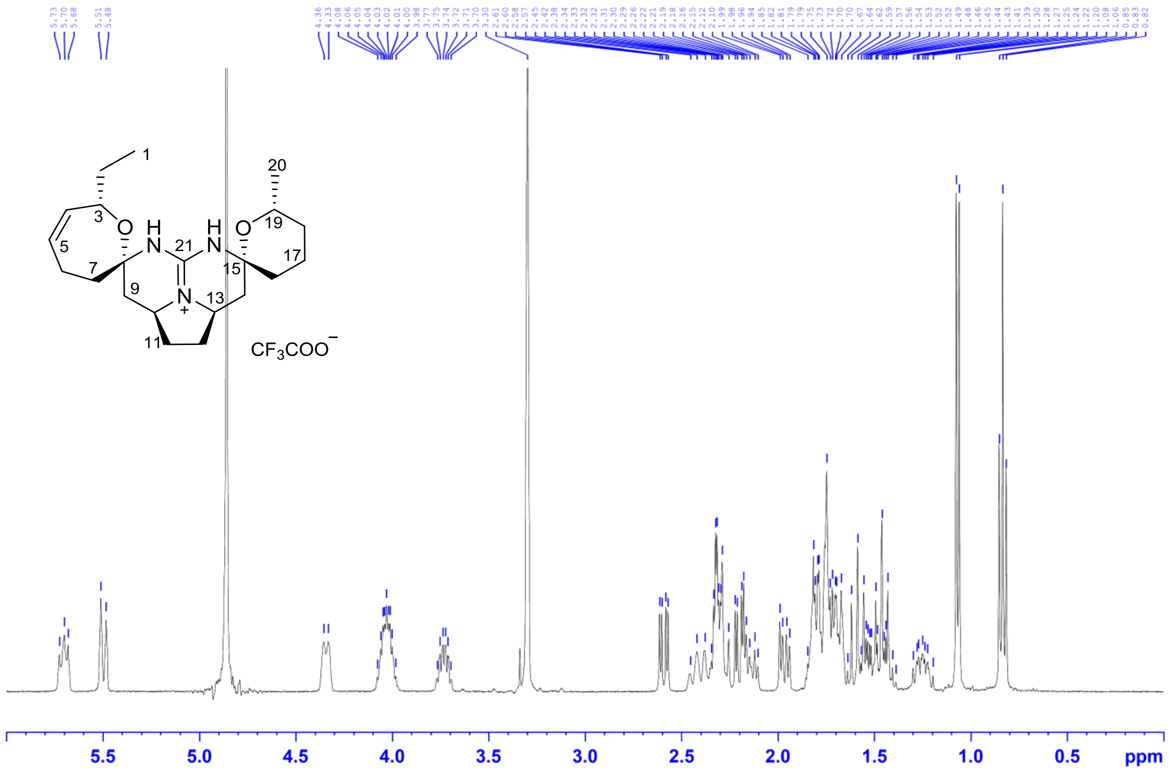

Figure S30: ^{1}H NMR spectrum **4** (400 MHz, CD_3OD).

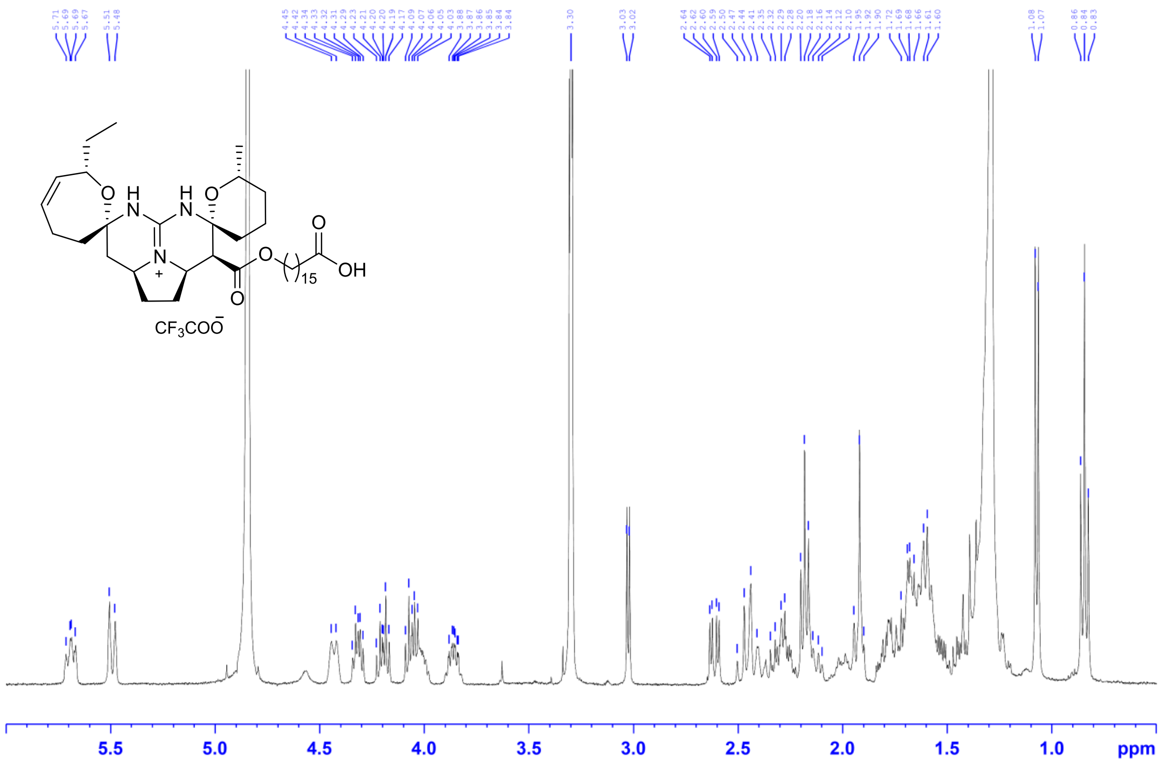

Figure S31: ^{1}H NMR spectrum **5** (400 MHz, CD_3OD).

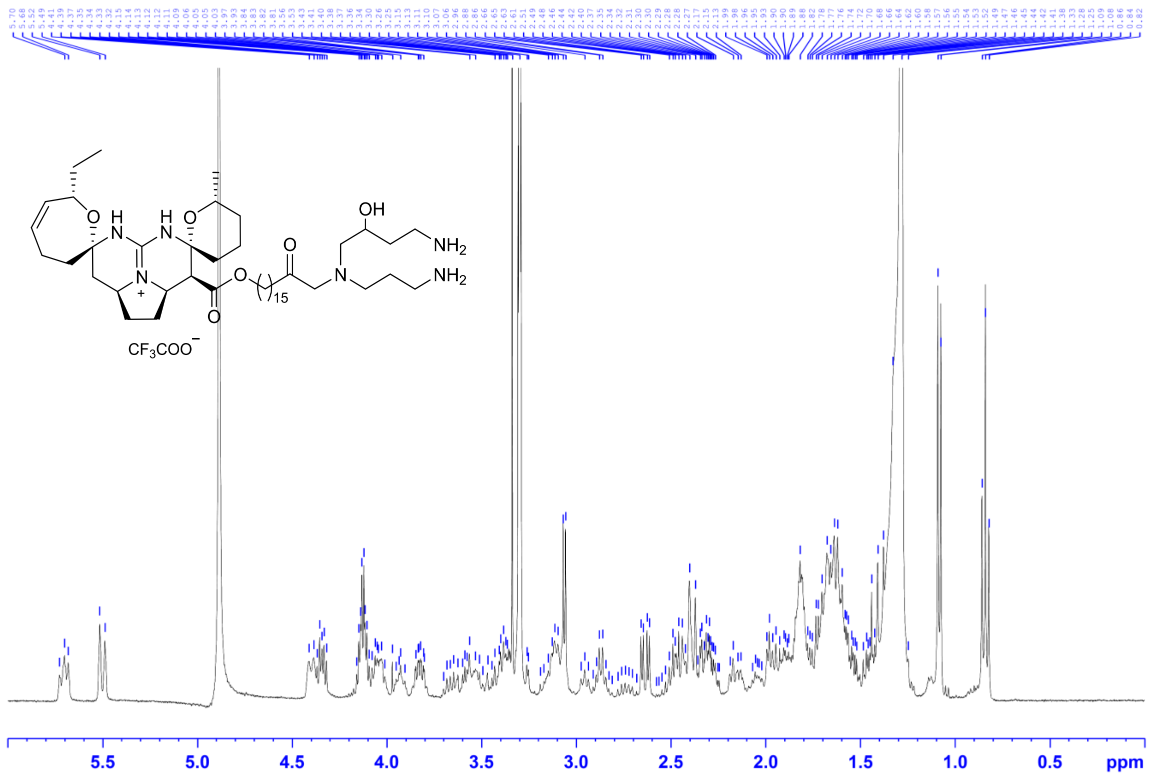

Figure S32: ^{1}H NMR spectrum **6** (400 MHz, CD_3OD).

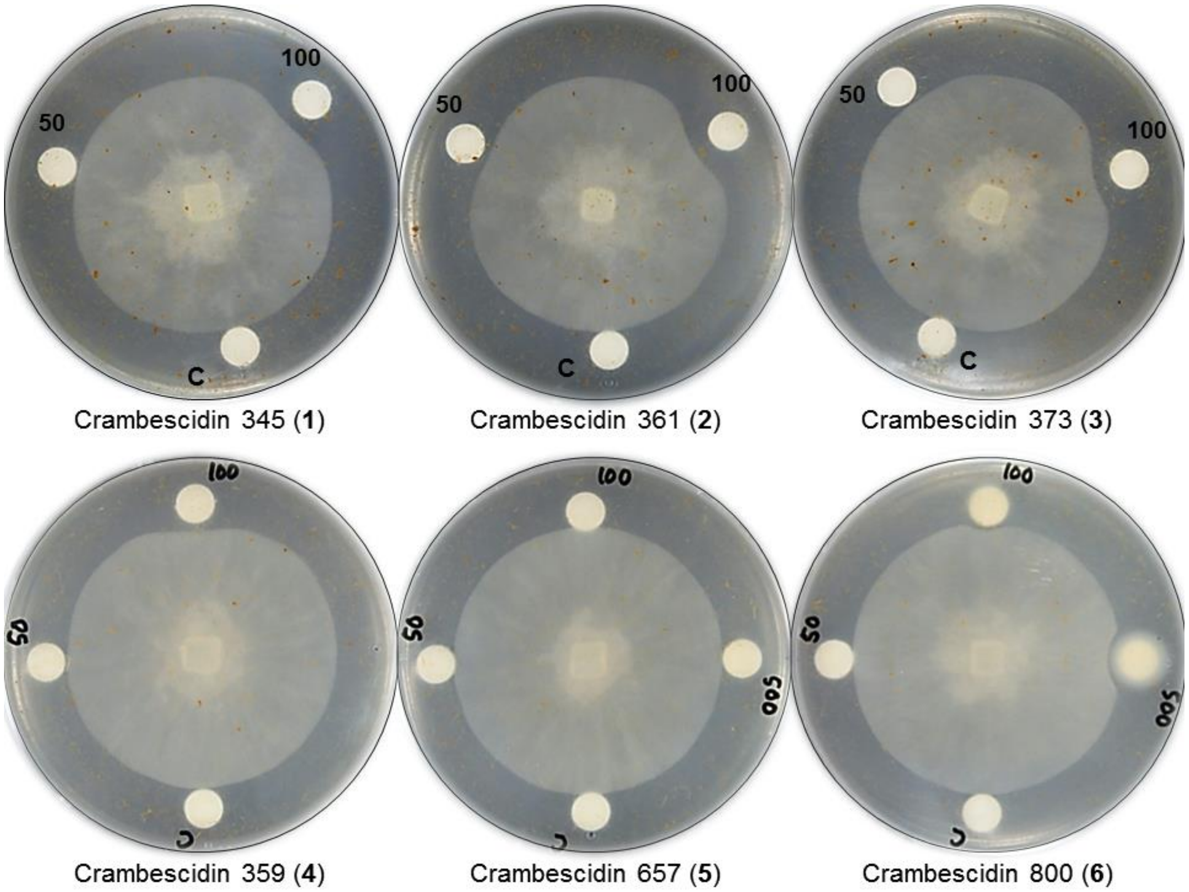

Figure S33: Anti-oomycete activity of **1–6**.
